# Treatment of latent tuberculosis infection in migrants in primary care *versus* secondary care

**DOI:** 10.1183/13993003.01733-2023

**Published:** 2024-11-07

**Authors:** Matthew Burman, Dominik Zenner, Andrew J. Copas, Lara Goscé, Hassan Haghparast-Bidgoli, Peter J. White, Vicky Hickson, Opal Greyson, Duncan Trathen, Richard Ashcroft, Adrian R. Martineau, Ibrahim Abubakar, Christopher J. Griffiths, Heinke Kunst

**Affiliations:** 1 Wolfson Institute of Population Health, Barts and The London School of Medicine and Dentistry, Queen Mary University of London, London, UK; 2 Homerton Healthcare NHS Foundation Trust, London, UK; 3 Institute for Global Health, University College London, London, UK; 4 MRC Centre for Global Infectious Disease Analysis and NIHR Health Protection Research Unit in Modelling and Health Economics, Department of Infectious Disease Epidemiology, Imperial College London, London, UK; 5 Modelling and Economics Unit, UK Health Security Agency, London, UK; 6 Newham Clinical Commissioning Group, London, UK; 7 City Law School, City, University of London, London, UK; 8 Blizard Institute, Barts and The London School of Medicine and Dentistry, Queen Mary University of London, London, UK; 9 Barts Health NHS Trust, London, UK

## Abstract

**Background:**

Control of latent tuberculosis infection (LTBI) is a priority in the World Health Organization strategy to eliminate TB. Many high-income, low TB incidence countries have prioritised LTBI screening and treatment in recent migrants. We tested whether a novel model of care, based entirely within primary care, was effective and safe compared to secondary care.

**Methods:**

This was a pragmatic cluster-randomised, parallel group, superiority trial (ClinicalTrials.gov: NCT03069807) conducted in 34 general practices in London, UK, comparing LTBI treatment in recent migrants in primary care to secondary care. The primary outcome was treatment completion, defined as taking ≥90% of antibiotic doses. Secondary outcomes included treatment acceptance, adherence, adverse effects, patient satisfaction, TB incidence and a cost-effectiveness analysis. Analyses were performed on an intention-to-treat basis.

**Results:**

Between September 2016 and May 2019, 362 recent migrants with LTBI were offered treatment and 276 accepted. Treatment completion was similar in primary and secondary care (82.6% *versus* 86.0%; adjusted OR (aOR) 0.64, 95% CI 0.31–1.29). There was no difference in drug-induced liver injury between primary and secondary care (0.7% *versus* 2.3%; aOR 0.29, 95% CI 0.03–2.84). Treatment acceptance was lower in primary care (65.2% (146/224) *versus* 94.2% (130/138); aOR 0.10, 95% CI 0.03–0.30). The estimated cost per patient completing treatment was lower in primary care, with an incremental saving of GBP 315.27 (95% CI 313.47–317.07).

**Conclusions:**

The treatment of LTBI in recent migrants within primary care does not result in higher rates of treatment completion but is safe and costs less when compared to secondary care.

## Shareable abstract

**This cluster-randomised control trial found that the treatment of latent tuberculosis infection in recent arrivals to the UK can be safely and effectively managed within primary care when compared to specialist secondary care services at lower cost.** https://bit.ly/4cmKzKm

## Graphical Abstract

**Figure:**
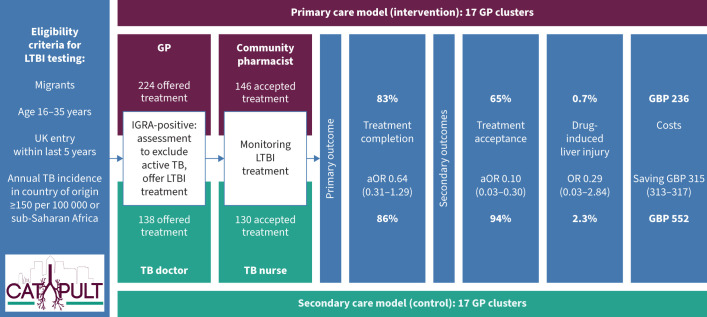
CATAPuLT (Completion and Acceptability of Treatment Across Primary care and the commUnity for Latent Tuberculosis) trial showing the eligibility criteria, trial design and results. 95% confidence intervals are indicated in brackets. LTBI: latent tuberculosis infection; TB: tuberculosis; GP: general practitioner; IGRA: interferon-γ release assay.

## Introduction

Tuberculosis (TB) is a leading global cause of death from infectious diseases [1]. The World Health Organization's (WHO) strategy to eliminate TB prioritises testing and treatment of those at high risk of latent TB infection (LTBI) [2]. It is estimated that approximately one quarter of the world's population (1.7 billion people) have LTBI and re-activation accounts for most active TB cases in low-incidence settings [3, 4]. Epidemiological modelling predicts that without the successful identification and treatment of LTBI in parallel to active case finding it will be impossible to achieve the WHO's target of TB elimination by 2050 [5].

There is a high prevalence of LTBI amongst recent migrants from high TB incidence countries and an increased risk of TB re-activation [6]. In many high-income, low TB incidence countries a high proportion of TB cases occur amongst migrants due to re-activation of LTBI [4], and many national TB control policies, including in England, have prioritised programmatic LTBI screening and treatment of recent migrants [7, 8]. Traditional models of care in many high-income countries are based on centralised assessment and treatment of patients with LTBI in specialist clinics by TB doctors and nurses, with low rates of treatment completion [8, 9].

There is limited evidence informing how to deliver LTBI treatment. A systematic review assessing interventions to improve adherence rates for LTBI found few studies related specifically to migrants [10]. To the best of our knowledge, no clinical trial has investigated the management of LTBI within primary care.

We hypothesised that a LTBI service within primary care would result in higher rates of LTBI treatment completion as care would be located closer to patients’ homes and use pharmacies that open outside of office hours and at weekends and would cost less. The CATAPuLT (Completion and Acceptability of Treatment Across Primary care and the commUnity for Latent Tuberculosis) trial compared the treatment of LTBI in primary care directly with treatment in specialist TB services within secondary care, the national model of care, to investigate the efficacy, safety and cost-effectiveness of this novel model of care [11].

## Methods

We conducted a pragmatic cluster-randomised, parallel group, superiority trial in 34 general practices in the London Borough of Newham, UK. We have previously reported the rationale and methods for the trial [12]. Clusters were individual general practices or groups of practices with a shared management structure providing primary medical care in Newham. Ethical approval was obtained from Camden and Kings Cross Research Ethics Committee (16/LO/0328).

The London Borough of Newham was a pilot site for the national LTBI screening programme for recent migrants. The borough introduced a novel model of care in which LTBI was treated entirely within primary care, the first time in the UK that LTBI has been managed programmatically outside a specialist TB service. General practitioners (GPs) offer screening with an interferon-γ release assay (IGRA) (QuantiFERON-TB Gold In-Tube; Cellestis, Chadstone, Australia). Patients with positive IGRA results are assessed by their GP, and if diagnosed with LTBI, offered treatment by a trained community pharmacist (supplementary material).

Individuals with LTBI were eligible if they were aged 16–35 years, from a country with a WHO-estimated annual TB incidence of ≥150 per 100 000 per year or from sub-Saharan Africa, and had entered the UK within the last 5 years, which is national policy [11]. The exclusion criteria were pregnancy, taking medications that could interact with LTBI treatment, evidence of infection with HIV or viral hepatitis B or C, known liver disease or cirrhosis, abnormal liver function tests (LFTs) prior to treatment, prior treatment for LTBI or active TB, inability to consent, requirement for LTBI treatment under direct supervision, or evidence of active TB.

Between October 2017 and April 2018, Newham piloted extended eligibility criteria to those who had been resident in the UK for up to 10 years, which was amended in the trial protocol.

### Intervention

GP practices were randomised to either the primary or secondary care arms. All patients diagnosed with LTBI were offered treatment for 3 months with Rifinah, a combination of rifampicin and isoniazid (Sanofi, Reading, UK), and pyridoxine, the recommended treatment in the UK [13]. In primary care (intervention), the GP assessed patients to exclude active TB and referred those with LTBI to a trained, accredited community pharmacist, selected by the patient, to initiate and monitor treatment. In secondary care (control), the GP referred IGRA-positive patients to the local TB service, where a TB doctor assessed the patients to exclude active TB and referred those with LTBI to a specialist TB nurse to initiate and monitor treatment.

In both arms the follow-up protocol required patients to attend for LFTs at 2–4 weeks and be reviewed monthly (supplementary material).

### Randomisation

A total of 34 general practices were randomised in four phases according to when they joined the trial (supplementary material). No practices withdrew between allocation and site initiation or during recruitment. At all phases and within all strata the randomisation was implemented through random permutation using Stata version 15 (StataCorp, College Station, TX, USA) and was conducted by the trial statistician (A.J. Copas).

### Outcomes

The primary outcome was treatment completion, defined as taking ≥90% of antibiotic doses assessed by prescription collection and pill count at the final review amongst those who accepted treatment. This threshold has been used in previous LTBI treatment trials [14]. If a patient attended without their medication, treatment completion was measured by self-report. Treatment completion data were missing for those patients that collected all prescriptions but did not attend a final visit.

Treatment acceptance was defined per protocol as the proportion of eligible patients who initiated treatment and attended TB clinics or community pharmacies on at least one occasion. During the implementation of the trial, an additional measurement was added post-hoc to assess for allocation bias, using aggregate data to assess the proportion of patients testing IGRA-positive during the period of trial recruitment that initiated treatment and attended TB clinics or community pharmacies on at least one occasion.

Adverse reactions were defined as any incident leading to discontinuation of LTBI treatment or hospitalisation, and included drug-induced liver injury (DILI). DILI was considered alanine transaminase (ALT) ≥3 times upper limit of normal (ULN) with symptoms or ALT ≥5 times ULN without symptoms. Separately, we assessed for adverse effects leading to discontinuation of treatment, excluding those with DILI.

The incidence of active TB occurring within 2 years of enrolment in the trial was calculated by linking the trial cohort to the national Enhanced TB Surveillance system hosted by Public Health England using deterministic and probabilistic matching [15].

Patient satisfaction was assessed as an ordinal outcome based on a single summary response item (score out of 10) in a non-validated treatment satisfaction questionnaire after 2 months of treatment (supplementary material).

### Blinding

The pragmatic design of the trial meant the allocation of patients and clusters. Data collection and analysis were not blinded.

### Statistical analysis

For calculation of sample size, we estimated that treatment completion would increase by 15% in the intervention arm (from 70% to 85%). We assumed an intracluster correlation coefficient (ICC) of 0.05 based on a previous LTBI screening study performed in East London [16], a two-sided α of 0.05 and 30% loss to follow-up or treatment non-acceptance. Based on 20 clusters (practices), we planned to recruit 1014 patients to provide 80% power (supplementary material). Lower than expected recruitment rates during the trial led the trial management group to recommend the recruitment of additional clusters to maintain statistical power, with a lower number of individual participants required. Based on a recalculated sample size using the original assumptions and including a coefficient of variation of cluster size of 0.5 to account for variability, we planned to recruit 442 patients from 34 clusters who accepted LTBI treatment.

We performed intention-to-treat analyses for all outcomes (supplementary material). For the main analysis of the primary outcome, missing data for patients who had been prescribed their final month of treatment but who failed to attend for final review were imputed. Multiple imputation by chained equations was conducted separately for each study arm [17]. A logistic regression model was used to impute the binary outcome, based on adherence in the preceding 2 months and reporting of moderate or severe adverse events at these reviews. We performed a sensitivity analysis for the primary outcome in which patients who failed to attend their final review were assumed not to have completed treatment. We analysed all outcomes using multilevel regression models (logistic, ordinal or Poisson) adjusted for sex, age, country/region of birth (ordinal), years in the UK (ordinal), number of patients managed by a general practice and number of individuals with a positive IGRA result at the practice at the time of randomisation, with the general practice as a random effect. We also performed unadjusted analyses for all outcomes as secondary effect measures. For health economic analyses, a decision-tree model was developed to compare the two strategies in terms of cost per LTBI case completing treatment following the standard National Institute for Health and Care Excellence reference case (supplementary material) [18]. All analyses were performed using Stata version 15. A p-value <0.05 was considered significant.

## Results

**FIGURE 1:**
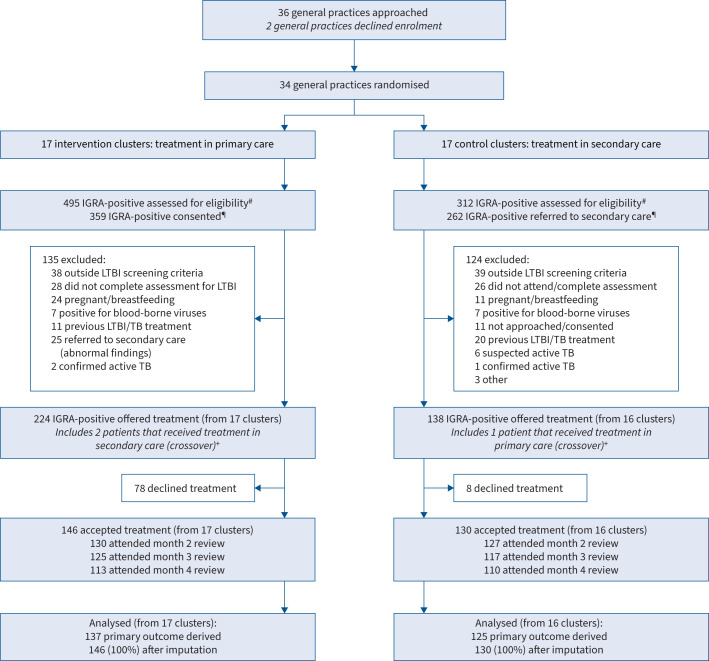
Trial profile. ^#^: practice-level data for trial recruitment period. ^¶^: in the intervention arm, patients were assessed and consented at a general practice. In the control arm, patients were referred to secondary care prior to assessment and consent. ^+^: patients allocated to an arm but receiving treatment in the other arm were considered to have crossed over and were analysed on an intention-to-treat basis. LTBI: latent tuberculosis infection; IGRA: interferon-γ release assay; TB: tuberculosis.

Between September 2016 and May 2019, 807 recent migrants tested IGRA-positive within the participating GP practice clusters in Newham. In the intervention arm (primary care), 495 patients tested IGRA-positive, 359 consented to share their data with the trial, 135 were excluded per protocol, 224 were offered treatment for LTBI and 146 patients accepted treatment for LTBI. In the control arm (secondary care), 312 patients tested IGRA-positive, 262 were referred to secondary care, 124 were excluded per protocol, 138 were offered treatment and 130 patients accepted treatment (figure 1). One GP practice cluster (secondary care arm) failed to recruit at least one patient who accepted treatment for LTBI.

The baseline characteristics of GP clusters were similar between arms (supplementary material). The baseline characteristics of trial participants, those who accepted treatment, were also similar between arms in terms of age, gender and country of origin. Most patients were from Southern Asia (India, Pakistan and Bangladesh) (80.1% intervention *versus* 83.9% control) and had been in the UK for <2 years (67.1% intervention *versus* 58.5% control) (table 1). For nine patients in the intervention group and five patients in the control group, the final review was missing and final outcomes were imputed.

**TABLE 1:** Baseline characteristics of participants accepting treatment for latent tuberculosis infection (n=276)

	Intervention (n=146)	Control (n=130)
**Age, years**	30 (26–33)	29.5 (26–33)
**Sex**		
Female	63 (43.2)	64 (49.2)
Male	83 (56.9)	66 (50.8)
**Country/region of birth**		
India	58 (39.7)	63 (48.5)
Bangladesh	37 (25.3)	28 (21.5)
Pakistan	22 (15.1)	18 (13.9)
Sub-Saharan Africa	22 (15.1)	9 (6.9)
Other	7 (4.8)	12 (9.2)
**Years in the UK**		
<2 years	98 (67.1)	76 (58.5)
2–4 years	19 (13.0)	26 (20.0)
>4 years	29 (19.9)	28 (21.5)

Data are presented as median (interquartile range) or n (%).

In primary care, after imputation, 82.6% of 146 patients accepting LTBI treatment completed it. In secondary care, 86.0% of 130 patients completed LTBI treatment. There was no significant difference in treatment completion between primary and secondary care (adjusted OR (aOR) 0.64, 95% CI 0.31–1.29). A sensitivity analysis, where failure to attend a final review was considered treatment failure, also found no difference in treatment completion between the two arms (76.7% *versus* 82.3% for primary care *versus* secondary care; aOR 0.53, 95% CI 0.23–1.22) (table 2). Similarly, excluding patients with missing data from the analysis found no difference in treatment completion between both arms (81.8% *versus* 85.6% for primary care *versus* secondary care; aOR 0.63, 95% CI 0.31–1.27). Amongst those who failed treatment, most (90.7% (39 out of 43)) did so because they did not attend to collect sufficient prescriptions to complete treatment before a final review, only 10.3% of patients (four out of 43) failed treatment because they had taken <90% of doses as assessed by pill count, or self-report at their final review. The ICC was 0 (1.2×10^−20^, 95% CI 0–0).

**TABLE 2:** Latent tuberculosis infection treatment completion, acceptance and patient satisfaction (n=362)

	Intervention	Control	OR (95% CI)	p-value	Adjusted OR (95% CI)	p-value
**Treatment completion**						
Missing data imputed	82.6	86.0	0.70 (0.35–1.38)	0.29	0.64 (0.31–1.29)	0.21
Without imputation, missing data excluded	81.8 (112/137)	85.6 (107/125)	0.69 (0.35–1.36)	0.28	0.63 (0.31–1.27)	0.20
Missing data considered failed	76.7 (112/146)	82.3 (107/130)	0.59 (0.27–1.30)	0.19	0.53 (0.23–1.22)	0.13
**Treatment acceptance**	65.2 (146/224)	94.2 (130/138)	0.10 (0.03–0.31)	<0.001	0.10 (0.03–0.30)	<0.001
**Patient satisfaction^#^**			1.84 (0.88–3.87)	0.11	1.80 (0.84–3.86)	0.13
≤7	3.1 (3/98)	2.5 (3/121)				
8	5.1 (5/98)	8.3 (10/121)				
9	14.3 (14/98)	27.3 (33/121)				
10	77.6 (76/98)	62.0 (75/121)				

Data are presented as % or % (n/N), unless otherwise stated. ^#^: score out of 10 (supplementary material), ordinal odds ratio. Odds ratios for ordinal outcomes based on proportional odds assumption.

Post-hoc, we assessed the marginal difference between treatment completion in primary care compared to secondary care amongst those patients with complete data. The adjusted difference in treatment completion was −6% (95% CI −15–3%) when comparing primary care to secondary care.

In the primary care arm, 224 patients were offered treatment for LTBI and 146 patients accepted it. In the secondary care arm, 138 patients were offered treatment and 130 patients accepted it. Treatment acceptance was lower in primary compared to secondary care (65.2% *versus* 94.2%; aOR 0.10, 95% CI 0.03–0.30) (table 2). Treatment acceptance was also assessed using aggregate data from GP surgeries, using a different denominator: those testing IGRA-positive during the period that a GP practice was recruiting to the trial. The proportion of all patients that tested IGRA-positive during the recruitment period that later accepted LTBI treatment within the trial was 29.5% (146 out of 495) in the primary care arm compared to 41.7% (130 out of 312) in the secondary care arm (figure 1). Treatment acceptance and completion varied between clusters in both the intervention and control arms. The mean (range) number of patients offered treatment at a GP surgery was 13.2 (2–30) in the primary care arm and 8.0 (0–32) in the secondary care arm. The mean (range) number of patients accepting LTBI treatment at a GP surgery was 8.6 (1–30) in the primary care arm and 7.6 (0–30) in the secondary care arm (supplementary material).

There were no hospitalisations in either arm. There were a low number of DILI events in both arms: 0.7% (one out of 146) in the primary care arm compared to 2.3% (three out of 130) in the secondary care arm (OR 0.29, 95% CI 0.03–2.84). In all cases where DILI occurred, LFTs normalised after treatment cessation. Two of the five patients who developed DILI subsequently went on to complete LTBI treatment (6 months isoniazid) outside the trial without further LFT derangement. The other three did not receive further treatment.

Other adverse effects leading to cessation of treatment, excluding DILI, occurred in the primary care arm in 4.1% (six out of 146) *versus* 6.2% (eight out of 130) in the secondary care arm (OR 0.65, 95% CI 0.22–1.94). Amongst those stopping treatment due to adverse effects, reported symptoms included nausea, vomiting, pruritus and rash (table 3).

**TABLE 3:** Adverse events (defined as any incident leading to discontinuation of treatment or hospitalisation)

	Intervention	Control	OR (95% CI)	p-value
**Serious adverse events leading to hospitalisation**	0	0		
**Adverse events**				
DILI (ATS criteria)^#^	0.7 (1/146)	2.3 (3/130)	0.29 (0.03–2.84)	0.29
DILI (local protocol)^¶^	0.7 (1/146)	3.1 (4/130)	0.22 (0.02–1.97)	0.18
Other adverse effects leading to discontinuation of treatment	4.1 (6/146)	6.2 (8/130)	0.65 (0.22–1.94)	0.44
Specific symptoms reported in those discontinuing treatment^+^				
Nausea	1.4 (2/146)	2.3 (3/130)		
Vomiting	0.0 (0/146)	0.8 (1/130)		
Itching	2.7 (4/146)	3.8 (5/130)		
Rash	1.4 (2/146)	0.0 (0/130)		
Paraesthesia	0.0 (0/146)	0.0 (0/130)		
“Flu-like” illness	0.0 (0/146)	0.0 (0/130)		
Abdominal pain/diarrhoea	0.0 (0/146)	0.0 (0/130)		

Data are presented as % or % (n/N), unless otherwise stated. DILI: drug-induced liver injury; ATS: American Thoracic Society; ALT: alanine transaminase; ULN: upper limit of normal. ^#^: DILI (ATS criteria): ALT ≥3 times ULN with symptoms or ALT ≥5 times ULN without symptoms; ^¶^: DILI (local protocol): ALT ≥2 times ULN leading to cessation of treatment; ^+^: excluding those who developed DILI.

There was no difference in patient satisfaction in the primary compared to the secondary care arm (aOR 1.80, 95% CI 0.84–3.86) (table 2).

There were no incident cases of TB in the trial cohort over a median (interquartile range) follow-up period of 1.9 (1.32–2.29 years). Three patients were identified with prevalent TB after testing for LTBI and were excluded from the trial.

The expected cost per treatment completed was GBP 236.43 (95% CI 235.83–237.03) in primary care and GBP 551.70 (95% CI 550.00–553.40) in secondary care, with an incremental saving of GBP 315.27 (95% CI 313.47–317.07).

## Discussion

This cluster-randomised control trial found that the proportion of recent migrants with LTBI who completed 3 months of rifampicin and isoniazid within primary care was not significantly higher than those managed in secondary care. The proportions of adverse events, including DILI, and satisfaction with care were similar in the two arms. LTBI treatment within primary care had substantially lower costs compared to secondary care. However, we found that fewer patients in primary care accepted treatment.

The LTBI treatment completion rates in our trial are similar to recent randomised controlled trials investigating short-course LTBI treatment amongst TB contacts [14, 19] and recent migrants [20], and higher than in three meta-analyses reporting on LTBI treatment outcomes in migrants [9, 21, 22]. Patients in our intervention arm were monitored in primary care by community pharmacists with high treatment completion rates which are similar to observational studies of LTBI treatment monitored by pharmacists in secondary care and support existing evidence for the role of community pharmacists in improving public health [23, 24].

The major limitation of our study is that we did not achieve our target sample size. Our original target recruitment rate per GP practice was based on aggregate data on positive IGRA results in the first year of the pilot programme, and did not include data on individual eligibility. Our study found that a large number patients were tested outside national and therefore trial eligibility criteria, such as age and year of entry to the UK. Steps were taken to mitigate this, including increasing the cluster number and extending the duration of the trial. Post-hoc analysis of the marginal difference in treatment completion (95% confidence intervals) suggests that the true difference could range from 15% lower to 3% higher in primary care when compared to secondary care.

Before randomisation, the number of individuals with positive IGRA tests was similar between arms. After randomisation, there were fewer individuals with positive IGRA tests in the secondary care arm. It is possible that a model of care where patients are referred to secondary care for treatment resulted in a change of behaviour at cluster level (GP practices) leading to fewer patients being tested, despite LTBI testing being a contractual obligation of GP practices in the region which was not altered by joining the trial (supplementary material). Since the demographic data on the patients who enrolled in the trial are similar in each arm and patients enrolled must be asymptomatic and identified by a positive IGRA test alone, the risk of bias in the comparison of treatment outcomes is low.

The primary outcome of the trial was to assess the completion of LTBI treatment delivered by community pharmacists and GPs (primary care) in direct comparison to specialist TB doctors and nurses (secondary care) and therefore compared patients that had accepted treatment. We identified lower rates of treatment acceptance within the primary care arm. This might be because of organisational factors such as the number of different sites patients had to visit for testing and treatment, or differences in clinical practice and/or confidence related to LTBI treatment between GPs and TB specialist doctors. Analysis of these factors was beyond the scope of the trial.

The impact of LTBI testing and treatment programmes on TB incidence amongst recent migrants is complex; a recent analysis in England, completed after our study, concluded that although there were low levels of participation nationally the programme provided a significant benefit to individuals, regardless of whether treatment was accepted or completed, because of early identification of prevalent active TB at the time of testing [25]. Early analysis of the programme in Newham found similar results with a high number of cases of prevalent active TB cases identified [26]. Although this trial found lower levels of acceptance for LTBI treatment using a primary care-based treatment model, aggregate data from the trial period suggest a secondary care treatment model may result in lower levels of LTBI testing (figure 1 and supplementary material). Since the analysis of the national programme suggests that individuals in a LTBI programme benefit once tested not just from treatment, this should be considered when interpreting the lower levels of treatment acceptance found in the primary care arm.

Our trial provides the first data directly comparing the treatment of recent migrants with LTBI by community pharmacists and GPs in primary care with specialist TB services in a high-income, low TB incidence country. The pragmatic design gives a realistic reflection of LTBI management by patients’ usual care providers in primary care and secondary care, enrolling the majority (73.4% (36 out of 49)) of GP practices, the sole providers of primary care, in the participating region.

Treatment completion was not significantly different in primary care; however, this finding should be interpreted with caution as we did not achieve our target sample size and there remains uncertainty if treatment completion in primary care is comparable to secondary care. Nevertheless, the trial provides the first results that suggest treatment in primary care could be a valid alternative to current models of care and be implemented within a new framework for TB control policy in low TB incidence countries. Our results are generalisable to the rest of the UK and other high-income, low TB incidence countries with similar health systems, including those within Europe, North America and Oceania [27].

We demonstrated that treatment for LTBI within primary care was associated with a large incremental saving per treatment completed when compared to treatment in secondary care without any impact on patient safety. WHO recommends a scale-up of TB preventive treatment to achieve TB eradication [28]. As the number of recent migrants with LTBI needed to treat to prevent one active TB case is high, national LTBI programmes must treat large numbers of individuals to reduce active TB incidence [6, 25]. Published data on LTBI screening and treatment programmes for recent migrants adopting a secondary care model show the majority of patients with LTBI do not complete treatment [25, 29]. Alternative models of care for LTBI which deliver effective care at low cost without utilising already sparse resources from existing TB services need to be found for LTBI migrant programmes to have an impact on TB control. Future research to develop a new framework could integrate a community-based model of care with migrant and culturally sensitive strategies to increase the uptake and completion of LTBI treatment [30].

The treatment of LTBI in recent migrants within primary care does not result in higher rates of treatment completion. It is safe and costs less but fewer patients accept treatment when compared to secondary care.

## Supplementary material

10.1183/13993003.01733-2023.Supp1 **Please note:** supplementary material is not edited by the Editorial Office, and is uploaded as it has been supplied by the author.
Supplementary material ERJ-01733-2023.Supplement

## Shareable PDF

10.1183/13993003.01733-2023.Shareable1 This one-page PDF can be shared freely online.
Shareable PDF ERJ-01733-2023.Shareable

## Data Availability

An anonymised version of the dataset can be made available upon request.
