# Supplementary material for: Treatment of latent tuberculosis infection in migrants in primary care versus secondary care
Source: Eur Respir J. 2024 Nov 7;64(5):2301733. doi: 10.1183/13993003.01733-2023 (PMC11540984; doi:10.1183/13993003.01733-2023)
Supplement: Supplementary file 1 [file ERJ-01733-2023.Supplement.pdf]

The treatment of latent tuberculosis infection in migrants in primary care versus secondary care

Online Data Supplement

Matthew Burman[1], Dominik Zenner[1], Andrew J Copas[2], Lara Goscé[2], Hassan Haghparast-Bidgoli[2], Peter J White[3,4], Vicky Hickson[1], Opal Greyson[1], Duncan Trathen[5], Richard Ashcroft[6], Adrian R Martineau[1], Ibrahim Abubakar[2], Christopher J Griffiths[1] and Heinke Kunst[7,8]

[1] Wolfson Institute of Population Health, Barts and The London School of Medicine and Dentistry, Queen Mary University of London, UK

[2] Institute for Global Health, University College London, London, UK

[3] MRC Centre for Global Infectious Disease Analysis, and NIHR Health Protection Research Unit in Modelling and Health Economics, Department of Infectious Disease Epidemiology, Imperial College London, UK

[4] Modelling & Economics Unit, UK Health Security Agency, London, UK

[5] Newham Clinical Commissioning Group, London, UK

[6] City Law School, City, University of London, UK.

[7] Blizard Institute, Barts and The London School of Medicine and Dentistry, Queen Mary University of London, UK

[8] Barts Health NHS Trust, London, UK

Corresponding author:

Dr. Heinke Kunst

Email h.kunst@qmul.ac.uk

Contents

Supplementary material for the CATAPuLT trial (Completion and Acceptability of Treatment Across Primary Care and the commUnity for Latent Tuberculosis) Page 1

Health Economic Analysis Methodology for the CATAPuLT trial (Completion and Acceptability of Treatment Across Primary Care and the commUnity for Latent Tuberculosis) Page 12

Appendix 1: CATAPuLT Trial Protocol

Appendix 2: CATAPuLT Trial Analysis Plan

Supplementary material for the CATAPuLT trial (Completion and Acceptability of Treatment Across Primary Care and the commUnity for Latent Tuberculosis)

Background

Unlike in many high-income countries, tuberculosis (TB) incidence in the United Kingdom (UK) increased over the latter part of the 20th Century, with TB rates increasing from the 1980s and reaching a peak in 2011. [1] Several interventions were made to reverse this trend. These included the introduction of pre-entry TB screening for those applying for visas to enter the UK from countries with a TB incidence greater than 40/100 000/year, and in 2015 the publication of the "Collaborative TB Strategy for England" by Public Health England and NHS England. [2] A key element of the strategy was the introduction of systematic screening for LTBI amongst recent arrivals from high TB incidence countries. It was proposed that recent migrants would be tested for latent tuberculosis infection (LTBI) within Primary care and those with positive results would be referred to secondary care (specialist TB clinics) for treatment. In the UK, care for patients with suspected latent or active TB is managed by specialist clinics within secondary care.

When the trial was planned the London borough of Newham had the highest incidence of TB in the United Kingdom (UK), with 86% of cases in 2014 occurring in those born outside the UK. [3] Since 2014, Newham has been a pilot site for the national LTBI screening programme for recent migrants.

The programme in Newham adopted a novel model of care for treating LTBI entirely based within primary care, the first time in the UK that LTBI has been managed programmatically outside a specialist TB service. GPs offer screening with an interferon gamma release assay (IGRA), QuantiFERON- TB Gold In-Tube (Cellestis, Australia). Patients with positive IGRA results are assessed, and, if diagnosed with LTBI, offered three months' treatment with rifampicin, isoniazid and pyridoxine monitored by a trained community pharmacist. As part of the programme, health care professionals in Newham received regular training on the diagnosis and management of LTBI. Outcome data from GP practices and community pharmacists was collated centrally by the Clinical Effectiveness Group (CEG, QMUL) for review by Newham Clinical Commissioning Group (CCG).

All GP practices within the borough were contractually obliged to offer this service which included how often a practice should try to contact eligible patients for testing, with payments made on a per patient basis for screening, completing testing, and clinical assessments to exclude active TB and discuss treatment. After the CATAPuLT trial completed, Newham CCG chose to continue to commission latent tuberculosis screening and treatment in recent migrants using this primary care-based model of care, factors in this decision included high rates of LTBI testing, the treatment outcomes within primary care, convenience for patients, the availability of resource within local secondary TB care services and lower costs. A summary of the national programme between 2015 and 2020 showed that Newham's activity accounted for approximately 10 percent of all tests completed nationally.[4]

Models of Care

The trial investigated an intervention within an existing LTBI screening programme. This programme offered migrants to the UK testing for LTBI if they met nationally set eligibility criteria (see Figure E1).

The precise care and follow required within each arm of the trial is explained in full in the trial protocol (see Appendix 1 sections 7.42 and 7.43).

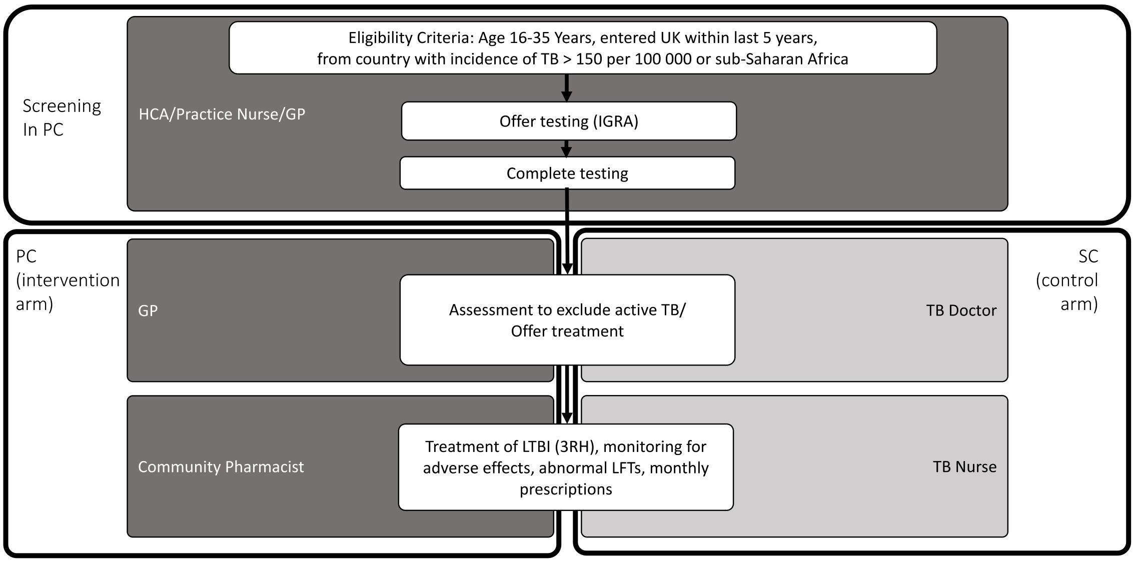

Figure E1: showing models of care within the CATAPuLT trial. Universal latent tuberculosis infection (LTBI) screening was offered to eligible recent migrants within primary. Primary Care Arm (PC): GP offered assessment to exclude active TB and offered LTBI treatment with onward referral to a community pharmacist to initiate and monitor treatment. Secondary Care arm (SC): TB doctor offered assessment to exclude active TB and LTBI treatment with referral to TB nurse to initiate and monitor treatment. PC: Primary Care, SC: secondary care, HCA: health care assistant, GP: General Practitioner, TB: tuberculosis, 3RH: 3 months of Rifampicin and Isoniazid with pyridoxine, LFTs: liver function tests.

Methodology

Randomisation

Phases one and two occurred in autumn 2016, and phases three and four occurred in autumn 2017. Randomisation in phase one was stratified by the number of IGRA positives and number of registered patients, in phase three by the number of IGRA positives, and in phases two and four was unrestricted due to small strata size

Additional Outcomes

Secondary outcomes within the trial protocol that were not included in the main manuscript are discussed here.

In addition to the primary outcome, treatment completion was also assessed as ordinal outcome using the categories <80%; 80-89·9%; or ≥90% doses taken. This was because there is no recognised standard for the proportion of prescribed doses completed that constitutes treatment completion. The other outcomes, definitions below, were treatment adherence and incidence of adverse events. These outcomes were defined for those individuals who started treatment.

Treatment adherence was assessed using prescription collection and point-of-care urine testing for metabolites of Isoniazid (Isoscreen®, UK). Adherence was classified into four categories: treatment stopped (at least one prescription missed); two or more negative urine tests (no isoniazid metabolites detected); one negative urine test; and no negative urine tests. Treatment adherence was also assessed using the Medication Adherence Report Scale (MARS-5).(4) Patients were asked to provide urine samples routinely at their month 2 and 3 reviews, or at their final review if there were concerns about adherence or they had failed to provide a urine sample at earlier reviews. We pre-specified that an analysis of adherence based on the MARS-5 questionnaire should only be performed if the measure had a bimodal distribution that would identify patients with lower adherence.

In addition to assessing Drug-induced liver injury (DILI) using American Thoracic Society (ATS) criteria (an ALT ≥3 times the upper limit of normal with symptoms or an ALT of ≥5 times the upper limit of normal without symptom), we assessed DILI based on pre-existing local protocol (an Alanine Aminotransferease (ALT) ≥2 times the upper limit of normal leading to cessation of treatment).

Table E1: Latent tuberculosis infection (LTBI) treatment completion (ordinal), adherence (ordinal) acceptance (n=362)

Outcome	Intervention % (N)	Control % (N)	OR (CI)	p	Adjusted OR (CI)	p
**Treatment completion (ordinal, missing data considered failed)**						
<80% of doses completed	23·3 (34/146)	15·4 (20/130)	0·57 (0·26-1·26)	0·17	0·51 (0·22-1·18)	0·12
80-89·9% of doses completed	0·0 (0/146)	2·3 (3/130)				
≥90% of doses completed	76·7 (112/146)	82·3 (107/130)				
**Adherence (Prescription collection and INH urine tests, ordinal OR)**						
Did not collect prescription	15·6 (19/122)	7·9 (10/127)	0·61 (0·31-1·19)	0·15	0·64 (0·32-1·28)	0·21
Two or more urine tests negative	0·8 (1/122)	2·4 (3/127)				
One negative urine test	7·4 (9/122)	6·3 (8/127)				
All urine tests positive	76·2 (93/122)	83·5 (106/127)				

Results

Treatment completion

Treatment completion was also assessed as an ordinal outcome: 76·7% in primary care (112/146) versus 82·6 % in secondary care (107/130) completed 90% or more of prescribed doses, no patient in primary care (0/146) versus 2·3% in secondary care (3/130) completed between 80 and 89·9% of prescribed doses, and 23·3% in primary care (34/146) versus 15·4% in secondary care (20/130) completed less than 80% of prescribed doses (ordinal aOR:0·51, 95%CI:0·22-1·18, table E1).

Our primary outcome was defined as taking at least 90% of antibiotic doses assessed by prescription collection and pill count at the final review among those who accepted treatment. We assessed how treatment failure was confirmed in our trial cohort (Table E2).

Most patients failed (90.7%, 39/43) because they had dropped out and not collected sufficient prescriptions to complete treatment before reaching a final review, only 4 of 43 patients who failed treatment did so because they had taken fewer than 90% of doses as assessed by pill count, or self-report at their final review. Treatment completion was assessed using pill-count at the final review in 80.7% of patients (92/114) in the primary care arm, and in 54.3% of patients in the secondary care arm (63/116).

Table E2: showing treatment outcomes for those with complete data.

	Primary care	Secondary care
Total with treatment completion data	137	125
Total completing treatment, n (%)	112 (81.8)	107 (85.6)
by pill-count	92	62
by self-report	21	51
Total failing treatment, n (%)	25 (18.3)	18 (14.4)
by failure to collect prescription	24	15
by pill-count	1	1
by self-report	0	2

Treatment completion (completing at least 90 percent of prescribed doses) was assessed at the final review by pill count unless the patient failed to attend with their medication, when completion was assessed by self-report. Failure to collect a prescription at an earlier review was considered treatment failure.

Adherence

We analysed adherence as an ordinal outcome combining prescription collection and a point of care urine test for metabolites of isoniazid (Isoscreen®, Oxfordshire, UK). We found no difference in treatment adherence between those treated in primary and secondary care (aOR 0·64 (95%CI:0·32-1·28 see table E1).

We found that, 76·2% of patients in primary care (93/122) versus 83·5% of patients in secondary care (106/127) collected all prescriptions, and all urine tests were positive; 7·4% in primary care (9/122) versus 6·3% of patients in secondary care (8/127) collected all prescriptions but had one negative urine test; 0·8% in primary care (1/122) versus 2·4% in secondary care (3/127) collected all prescriptions but had two or more negative urine tests; and 15·6% (19/122) in primary care versus 7·9% in secondary care (10/127) failed to collect one prescription.

Medication Adherence Report Scale 5 (MARS5)

Treatment adherence was also assessed using the five-point adherence questionnaire (MARS5 tool). This has not been validated in patients being treated for LTBI. Patients were asked to complete the MARS5 at each of the 3 follow up visits. As per the analysis plan, MARS5 scores were reviewed to assess their distribution, specifically whether greater than 95 percent of the scores were greater than 90 percent of the maximum score e.g. 23 or more out of 25 and whether the distribution was bimodal. The distribution of scores after each month of treatment was heavily skewed with a unimodal distribution (see figure E2). Adherence as assessed by the MARS5 scale was therefore not formally analysed due to a lack of bimodal distribution.

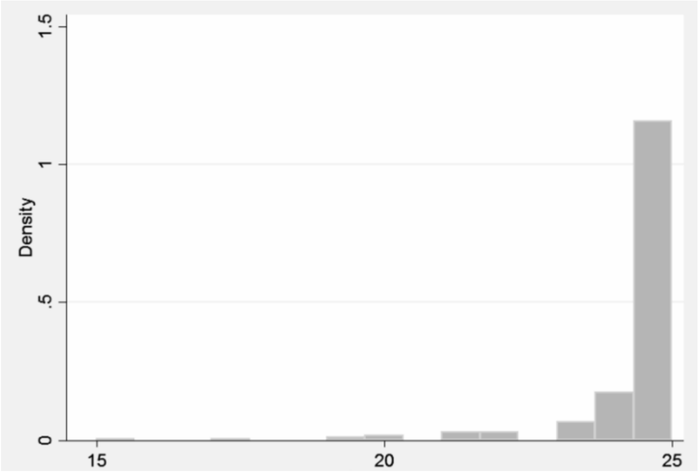

a. MARS5 score after 1 month of treatment

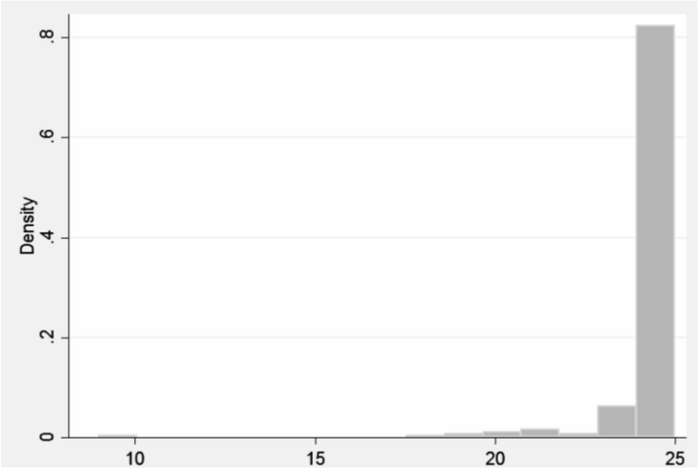

b. MARS5 score after 2 months of treatment

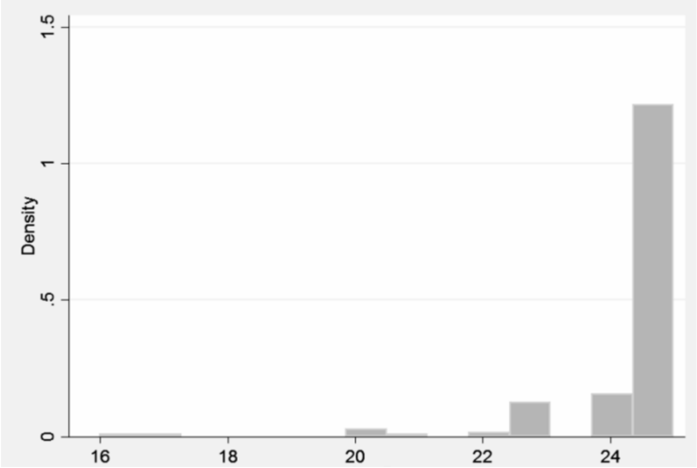

c. MARS5 score after 3 months of treatment

Figure E2: showing histograms for MARS5 scores after 1, 2 and 3 months of treatment for latent tuberculosis infection (LTBI). Scores out of 25. MARS5: Medication Adherence Report Scale 5

After one month of treatment, fewer than 95 percent of scores were ≥23 out of 25. After two months of treatment, fewer than 95 percent of scores were ≥23 out of 25, after three months of treatment, greater than 95 percent of scores were less than≥23 out of 25.

Based on the distribution of the MARS5 data, we did not perform any statistical analysis comparing MARS5 scores between the two arms. Below the MARS5 results are summarised including comparison with the primary outcome of treatment completion.

We compared MARS5 scores by month. After one month of treatment 10.1% of patients (13/129) in the primary care arm reported a MARS5 score suggestive of poor adherence compared to 3.3% of patients in the secondary care arm (4/120). After two months of treatment, 8.0% (10/125) of patients in the primary care arm and 2.6% (2/114) in the secondary care arm reported a MARS5 score suggestive of poor adherence. After three months of treatment, 6.2% (7/113) of patients in the primary care arm and 1.8 % of patients (2/110) patients in the secondary care arm reported a MARS5 score suggestive of poor adherence (see Table E3).

Table E3: Adherence based on Medication Adherence Report Scale 5 (MARS5) scores at each month review in primary and secondary care

Treatment Reviews	Adherence (MARS5 score)	Primary care % (n)	Secondary care % (n)
Post one month of treatment	Poor (≤22)	10.08 (13/129)	3.33 (4/120)
	Good (≥23)	89.92 (116/129)	96.47 (116/120)
Post two months of treatment	Poor (≤22)	8.00 (10/125)	2.63 (3/114)
	Good (≥23)	92.00 (115/125)	97.37 (111/114)
Post three months of treatment	Poor (≤22)	6.19 (7/113)	1.82 (2/110)
	Good (≥23)	93.81(106/113)	98.18 (110)

Scores ≤22: poor adherence, scores ≥23: good adherence.

We compared MARS5 scores, where available to the primary outcome of treatment completion. After one month of treatment, amongst the patients, who had a MARS5 score recorded, who later failed treatment, based on completing less than 90 percent of prescribed does, one out of 17 reported a MARS5 score suggestive of poor adherence (≤22). After two months of treatment, amongst the patients who later failed treatment, one of 8 reported a MARS5 score suggestive of poor adherence. After three months of treatment, among the patients who failed treatment, one out of 5 reported a MARS5 score suggestive of poor adherence (see Table E4).

Table E4 Medication Adherence Report Scale 5 (MARS5 score by month) post-treatment and treatment outcome amongst those patients accepting treatment without missing data at their final review (n=262).

	Post one month of treatment			Post two months of treatment			Post three months of treatment		
Treatment outcome	MARS5≤22	MARS5≥23	missing	MARS5≤22	MARS5≥23	missing	MARS5≤22	MARS5≥23	missing
failed	1	16	26	1	7	35	1	4	38
completed	16	203	0	11	206	2	8	210	1

Scores ≤22: poor adherence, scores ≥23: good adherence.

We found that the MARS5 tool was not useful for assessing adherence in the treatment of LTBI. Low MARS5 scores did not correlate with treatment failure or other measures of adherence.

Drug Induced Liver Injury

Using local protocols, 0·7% (1/146) of patients developed a DILI in the PC arm compared to 3·1% (4/130) in the SC arm (OR:0·22, 95%CI:0·02-1·97) (see Table E5)

Table E5: Drug induced liver injury (DILI) based on local protocol

Outcome	Intervention	Control	OR (CI)	p

	% (N)	% (N)		
Drug-induced liver injury (local protocol)	0·7 (1/146)	3·1 (4/130)	0·22 (0·02-1·97)	0·18

DILI (local protocol): an ALT more than two-times the upper limit of normal leading to cessation of treatment

Cluster information

A total of 34 GP clusters were recruited to the trial. The mean number of listed patients at GP surgeries at the time of randomisation was 7882 (range 4000-14000) in the primary care arm and 8824 (range 2000-21000) in the secondary care arm. The mean number of IGRA positive tests recorded at GP practices at the time of randomisation was 14.8 (range 4-38) in the primary care arm and 13.4 (range 0-45) in the secondary care arm.

During the period of trial recruitment, the mean number of patients offered testing at GP practices in the primary care arm was 262 compared to 178 in the secondary care arm. The mean number of patients tested per GP cluster was 129 in primary care compared to 84 in secondary care. The proportion of patients offered testing who completed it was 47 percent in primary care and 49 percent in secondary care and the proportion testing IGRA positive was 21.8 versus 22.6 respectively. The mean number of IGRA positive results per GP practice during trial recruitment was 29.1 in primary care and 18.4 in secondary care.

During the trial the mean number of patients offered treatment at a GP surgery was 13.2 (range 2-30) in the primary care arm and 8.0 (range 0-32) in the secondary care arm. The mean number of patients accepting LTBI treatment at a GP surgery was 8.6 (1-30) in the primary care arm and 7.6 (0-30) in the secondary care arm (see table E6). The full characteristics of each GP cluster are shown in Table E7.

Table E6

a) Baseline summary characteristics of GP surgery clusters in the CATAPULT trial.

		Intervention (Primary Care)	Control (Secondary Care)
Baseline GP cluster characteristics (randomisation strata)	patients listed at general practice mean (range)	7882 (4000-14000)	8824 (2000-21000)
	IGRA positive prior to joining trial mean (range)	14.8 (4-38)	13.4 (0-45)
GP cluster data	Offered treatment mean (range)	13.2 (2-30)	8.0 (0-32)
	Accepting treatment mean (range)	8.6 (1-30)	7.6 (0-30)

b) Aggregate testing data for GP clusters during trial recruitment period (source CEG)

		Intervention (Primary Care)	Control (Secondary Care)
Aggregate GP cluster data during trial recruitment period	Patients offered testing per cluster, mean (range)	262 (14-1108)	178 (15-520)
	Patients tested per cluster, mean (range)	129 (10-263)	84 (4-272)
	Mean IGRA positive results per cluster	29.1 (0-70)	18.4 (5-73)
	Proportion offered testing that completed % (total tests completed/total tests offered)	47.1 (2193/4462)	49.1 (1431/3033)
	Proportion testing IGRA positive % (total positive/ total tests completed)	21.8 (312/1431)	22.6 (495/2195)

GP: General Practitioner, IGRA: interferon gamma release assay, CEG: Clinical Effectiveness Group, Queen Mary University of London

Table E7: showing baseline characteristics, number of patients offered, accepting and completing latent tuberculosis infection (LTBI) treatment by GP cluster. (Clusters 1-17: Secondary Care, Clusters 18-34: Primary Care).

		Baseline characteristics (Randomisation strata)		Trial data					
**Arm**	**Cluster**	**Total patients listed at general practice**	**Total IGRA positive prior to joining trial**	**Offered treatment (n)**	**Accepting treatment (n)**	**Accepting treatment (%)**	**Completing treatment (n)**	**Completing treatment (%)**
Control (Secondary Care)	1	9000	10	3	2	66.7	2	100
	2	10000	18	13	13	100	12	92.3
	3	7000	7	2	2	100	1	50
	4	7000	26	9	9	100	9	100
	5	2000	22	32	30	93.8	23	76.7
	6	4000	7	3	3	100	3	100
	7	4000	8	15	15	100	14	93.3
	8	3000	4	3	3	100	3	100
	9	21000	33	3	2	66.7	1	50
	10	13000	45	29	29	100	19	65.5
	11	8000	16	9	9	100	8	88.9
	12	8000	6	1	1	100	1	100
	13	9000	0	0	0	NA	0	NA
	14	7000	5	1	1	100	1	100
	15	6000	7	4	4	100	4	100
	16	15000	10	5	4	80	3	75
	17	17000	3	4	3	75	3	100
Intervention (Primary Care)	18	10000	27	12	8	66.7	5	62.5
	19	6000	16	30	30	100	24	80
	20	13000	15	5	5	100	4	80
	21	7000	5	4	1	25	1	100
	22	14000	11	17	6	35.3	4	66.7
	23	4000	13	6	5	83.3	5	100
	24	4000	4	11	7	63.6	3	42.9
	25	4000	16	12	1	8.3	1	100
	26	10000	22	19	12	63.2	8	66.7

	27	5000	38	26	20	76.9	17	85
	28	12000	21	25	14	56	10	71.4
	29	4000	10	3	2	66.7	2	100
	30	9000	12	10	8	80	4	50
	31	7000	5	6	2	33.3	2	100
	32	12000	15	28	18	64.3	16	88.9
	33	4000	10	2	1	50	0	0
	34	9000	11	8	6	75	6	100

Health Economic Analysis Methodology for the CATAPuLT trial (Completion and Acceptability of Treatment Across Primary Care and the commUnity for Latent Tuberculosis)

A cost-effectiveness analysis was performed by calculating the incremental cost per patient completing treatment in primary care (intervention) compared to secondary care (control).

Methods

The analysis was conducted from an NHS perspective with a two-year time horizon and standard discounting 3·5% per annum was applied. The decision analysis model described the possible pathways of patients at the different time point of adherence control under each strategy. Estimates of costs were obtained from the NHS Reference Costs[5] and sources included in the local LTBI service specification. For each arm the estimated cost per patient completing treatment was calculated by dividing the costs incurred for all patients in the relevant arm by the number completing treatment and then the arms were compared incrementally. A sensitivity analysis was conducted to evaluate uncertainty around costs and probabilities of progression along pathways to treatment completion.

Decision Tree

We used a decision tree to model the latent tuberculosis infection (LTBI) treatment pathway for recent migrants (see Figure E3).

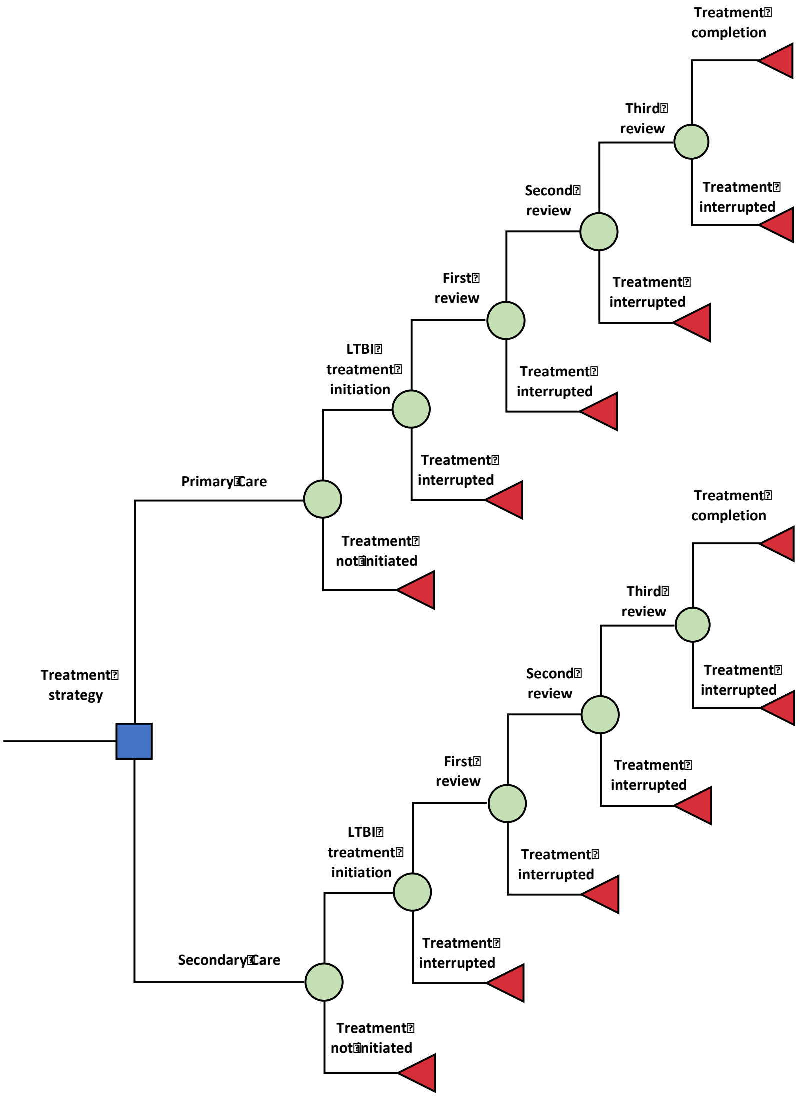

Figure E3. Latent tuberculosis treatment decision tree schematic. The two branches represent the two different care pathways: primary and secondary care. The cost-effectiveness of the intervention was studied comparing the cost per treatment completion in the two arms

Costs

Primary care cost data were collected during the study period based on the existing service specification for the Newham Care Commissioning Group (CCG) LTBI extended primary care service specification (see Table E8). NHS tariffs were used for secondary care costs (see Table E9). Pre-treatment investigation costs included all those required as per the CATAPuLT study protocol and local practice: including baseline full blood count, urea and electrolytes, liver function tests (LFTs), C-reactive protein, Hepatitis B virus, Hepatitis C virus and Human Immunodeficiency virus serology and a chest radiograph (CXR). All investigations and treatment costs are included within the NHS tariff for each visit.

Table E8: Primary care cost data (2019 GBP)*

Description	***Value***
Tests pre-treatment: blood tests (including FBC, U+E, LFT, HIV, HBV, HCV serology) + CXR	£74.23
GP assessment of IGRA-positive individuals: review by GP, offer of treatment, initiation of LTBI treatment and prescription	£55.67
Community Pharmacist: professional + administration fees per treatment initiation and monthly pharmacy review (total)	£75.00
Comprising	
Professional + administration fees per treatment initiation	£30.00
Professional + administration fees 1st review	£25.00
Professional + administration fees 2nd review	£15.00
Professional + administration fees 3rd review	£5.00
Drug costs (per month)	£25.55
Urine test + administration (per visit)	£12.00
LFT after initiation of treatment	£4.00

*Source: Newham CCG LTBI extended primary care service specification (available on request 2018-2019).
CCG: clinical commissioning group, CRP: C-reactive protein, CXR: chest radiography, FBC: Full blood count, GBP: British pound sterling, GP: general practitioner, HBV: Hepatitis B virus, HCV: Hepatitis C virus, HIV: Human Immunodeficiency virus serology, IGRA: Interferon Gamma Release Assay, LFT: liver function tests, LTBI: Latent tuberculosis infection, U+E: Urea and electrolytes, Urine Test: point of care urine test for metabolites of isoniazid (Isoscreen®, Oxfordshire, UK).

Table E9: Secondary care costs data*

Description	*Value*
**NHS tariff per single respiratory medicine clinic visit - first attendance	£215.00
**NHS tariff per single respiratory medicine clinic visit - follow-up visit	£96.00

***Source**: NHS National Tariff Workbook (https://www.england.nhs.uk/publication/2019-20-national-cost-collection-data-publication/) 2019-2020. ** Tariff includes costs for any investigations including blood tests, chest radiographs and treatment.

Outcome data

Outcome data from the intervention and control arms (see Table E10) the numbers of people who attended monthly reviews during the 3-months duration treatment and the final figure of treatments completed.

Table E10: The total number of patients retained in each arm of the trial at different time points during their latent tuberculosis infection (LTBI) treatment. The variability around these proportions has been estimated using the svy mode in STATA 15

	Primary care		***Secondary care***	
Description	***Total (n)***	***Proportion (%) (95% CI)***	***Total***	***Proportion (%) (95% CI)***
Patients eligible for treatment	224	N/A	138	N/A
Patients initiating treatment	146	65.2 (50.1-77.3)	130	94.2 (88.0-97.3)
Patients who had LFTs performed	122	83.6 (75.6-89.3)	130	100.0 (100.0-100.0)
Patients attending for 1st review	130	89.0 (81.2-93.9)	127	97.7 (91.7-99.4)
Patients attending for 2nd review	125	96.2 (93.5-97.7)	117	92.1 (86.4-95.6)
Patients attending for 3rd review	113	90.4 (81.0-95.4)	110	94.0 (84.6-97.8)
Patients completing treatment	112	99.1 (92.9-99.9)	107	97.3 (91.7-99.1)

Sensitivity analysis

The data presented in tables E8, E9 and E10 was used to parametrise the decision-tree model. Probabilistic sensitivity analysis was then performed to assess the effect of variation in the expected and incremental costs with respect to cost and probability data. A Monte Carlo simulation with 1000 iterations was performed, assuming a gamma distribution with 20% uncertainty for costs, and beta distribution for probabilities with variability calculated on proportions of patients at each step of the model (see Table E10).

Results

The analysis showed that the expected cost per treatment completed was £236.43(95%CI:£235.83-£237.03) in primary care and £551.70(£550.00-£553.40) in secondary care. This resulted in an incremental saving of £315. 27(£313.47-£317.07) in primary care compared to secondary care arm (see main manuscript).

References

1. Thomas HL, Harris RJ, Muzyamba MC, Davidson JA, Lalor MK, Campbell CNJ, Anderson SR, Zenner D: **Reduction in tuberculosis incidence in the UK from 2011 to 2015: a population-based study**. *Thorax epub ahead of print* 2018:thoraxjnl-2017-211074-211077.
2. Collaborative Tuberculosis Strategy for England 2015 to 2020. Public Health England/NHS England. 2015. https://assets.publishing.service.gov.uk/government/uploads/system/uploads/attachment_data/file/403231/Collaborative_TB_Strategy_for_England_2015_2020_.pdf. Accessed 04/04/2017.
3. Loutet MG, Burman M, Jayasekera N, Trathen D, Dart S, Kunst H, Zenner D: **National roll-out of latent tuberculosis testing and treatment for new migrants in England: a retrospective evaluation in a high-incidence area**. *European Respiratory Journal* 2018, **51**(1):1701226.
4. Latent TB infection testing and treatment programme for migrants. Presenting data between 1 April 2015 to 31 March 2020. Public Health England. https://assets.publishing.service.gov.uk/media/6046616ad3bf7f1d1420700c/LTBI-testing-and-treatment_2019_to_2020.pdf. Accessed 10/10/2023.
5. **NHS Reference Costs 2019-2020.** [https://www.england.nhs.uk/publication/2019-20-national-cost-collection-data-publication/]

Appendix 1

Can latent tuberculosis infection in recent migrants be treated effectively and safely in primary care? A cluster randomised controlled trial

**Short Title/Acronym**	**Completion and Acceptability of Treatment Across Primary Care and The Community for Latent Tuberculosis (CATAPULT)**
**Sponsor**	*Queen Mary, University of London*<br><br>*Contact person of the above sponsor organisations is:*<br><br>**Dr Sally Burtles<br>Director of Research Services and Business Development<br>Queen Mary University of London<br>Joint Research Management Office (JRMO)<br>Queen Mary Innovation Centre<br>Lower Ground Floor<br>5 Walden Street<br>London, E1 2EF<br>Tel: 020 7882 7260<br>Office fax: 020 7882 7276 (Internal: 13-7276)<br>E-mail: sponsorsrep@bartshealth.nhs.uk**
**REC Reference**	
**Chief Investigator**	**Dr. Heinke Kunst<br>Senior Lecturer/<br>Consultant in Respiratory Medicine<br>Telephone no: +447966285212<br>Email: h.kunst@qmul.ac.uk<br>Work address: The Blizard Institute, Barts and the London School of Medicine and Dentistry, Queen Mary, University of London.**

Protocol development

Dr Matthew Burman
Research Fellow/Registrar
Telephone no: +447779710285
m.burman@qmul.ac.uk
Work address: Centre for Primary Care & Public Health, Yvonne Carter Building, Blizard Institute, Queen Mary University of London, 58 Turner Street, London, E1 2AB

Dr Dominik Zenner

Local Collaborator
Consultant Epidemiologist and General Practitioner
Telephone contact no: 02083277456
Email: Dominik.Zenner@phe.gov.uk
Work address: Respiratory Diseases Department, Centre for Infectious Disease Surveillance and Control, Public Health England, London, NW9 5EQ

Other Collaborators/Trial Steering Committee

Professor Chris Griffiths
Professor of Primary Care
Tel: 0207 882 2509
Email: c.j.griffiths@qmul.ac.uk
Work address: Centre for Primary Care & Public Health, Yvonne Carter Building, Blizard Institute, Queen Mary University of London, 58 Turner Street, London, E1 2AB

Professor Ibrahim Abubakar
Professor of Infectious Disease Epidemiology
Tel: 0207 679 0954
Email: i.abubakar@ucl.ac.uk
Work address: Research Department of Infection and Population Health Mortimer Market Centre, Capper Street, University College London, London, WC1E 6JB

Professor Adrian Martineau
Clinical Professor of Respiratory Infection and Immunity
Tel: 020 7882 2551
Email: a.martineau@qmul.ac.uk
Work address: Centre for Primary Care & Public Health, Yvonne Carter Building, Blizard Institute, Queen Mary University of London, 58 Turner Street, London, E1 2AB

Professor Richard Ashcroft
Professor of Bioethics
Email: r.ashcroft@qmul.ac.uk
Work address: School of Law, Queen Mary, University of London, Mile End Road, London, E1 4NS

Trial Statistician

Dr Andrew Copas
Reader in Statistics Infection & Population Health
Tel: 020 3108 2062
a.copas@ucl.ac.uk
Work address: Research Department of Infection & Population Health, The Mortimer Market Centre, Capper St, University College London, London, WC1E 6JB

The study will take place in GP Practices under the remit of Newham CCG

Contents Page

1. GLOSSARY OF TERMS AND ABBREVIATIONS
2. SIGNATURE PAGE
3. SUMMARY/SYNOPSIS
4. INTRODUCTION
5. TRIAL OBJECTIVES
6. METHODOLOGY
   Inclusion Criteria
   Exclusion Criteria
   Study Design / Plan – Study Visits
   Study Scheme Diagram
7. STUDY PROCEDURES
   Schedule of Assessment (in Diagrammatic Format)
   End of Study Definition
8. STATISTICAL CONSIDERATIONS
9. ETHICS
10. DATA HANDLING AND RECORD KEEPING
    Confidentiality
    Record Retention and Archiving
11. LABORATORIES
12. TOOLS
13. SAFETY REPORTING
14. MONITORING &AUDITING
15. FINANCE AND FUNDING
16. INDEMNITY
17. DISSEMINATION OF RESEARCH FINDINGS
18. REFERENCES
19. APPENDICES

1. GLOSSARY of Terms and Abbreviations

AE	Adverse Event
AR	Adverse Reaction
ASR	Annual Safety Report
CA	Competent Authority
CI	Chief Investigator
CRF	Case Report Form
CRO	Contract Research Organisation
DMC	Data Monitoring Committee
EC	European Commission
GAfREC	Governance Arrangements for NHS Research Ethics Committees
ICF	Informed Consent Form
JRMO	Joint Research Management Office
LTBI	Latent Tuberculosis Infection
NHS REC	National Health Service Research Ethics Committee
NHS R&D	National Health Service Research & Development
Participant	An individual who takes part in a clinical trial
PI	Principal Investigator
PIS	Participant Information Sheet
QA	Quality Assurance
QC	Quality Control
RCT	Randomised Controlled Trial
REC	Research Ethics Committee
SAE	Serious Adverse Event
SDV	Source Document Verification
SOP	Standard Operating Procedure
SSA	Site Specific Assessment
TB	Tuberculosis
TMG	Trial Management Group
TSC	Trial Steering Committee

2. SIGNATURE PAGE

Chief Investigator Agreement

The clinical study as detailed within this research protocol **(Version 7, dated 1 May 2019)**, or any subsequent amendments will be conducted in accordance with the Research Governance Framework for Health & Social Care (2005), the World Medical Association Declaration of Helsinki (1996) and the current applicable regulatory requirements and any subsequent amendments of the appropriate regulations.

Chief Investigator Name: Heinke Kunst

Chief Investigator Site: Queen Mary, University of London

Signature and Date: 01/05/19

Heinke Kunst

3. SUMMARY/SYNOPSIS

**Short Title**	*CATAPULT (Completion and Acceptability of Treatment Across Primary care and The commUnity for Latent Tuberculosis)*
**Methodology**	*Cluster-randomised trial evaluating treatment completion (the primary outcome), uptake, acceptability, safety and cost-effectiveness of treating latent tuberculosis infection (LTBI) in migrants in primary care, compared with secondary care*
**Research Sites**	*TB clinic Shrewsbury Road (Newham Hospital) and GP surgeries in Newham*
**Objectives/Aims**	***1. Aims and Objectives***<br>***1.1. Primary objective***<br>• *To determine whether an innovative programme of treating latent tuberculosis infection (LTBI) in primary care increases LTBI treatment completion (defined as taking at least 90% of antibiotic dosages based on pill count) compared with current practice (treatment in secondary care).*<br><br>***1.2. Secondary objectives***<br>• *To assess the proportion of patients completing greater than 80% and 85% of antibiotic dosages based on pill count.*<br>• *To assess the proportion of patients who are adherent to treatment based on the MARS5 tool, prescription collection and point-of-care urine testing for metabolites of Isoniazid.*<br>• *To describe the proportion of individuals in the two treatment arms who accept LTBI treatment.*<br>• *To assess the incidence of adverse effects of treatment for LTBI including drug induced liver injury (DILI) in both arms.*<br>• *To assess rates of active tuberculosis (TB) cases in primary and secondary care during and after the study period measured as case notifications to Public Health England (PHE) using the Enhanced TB surveillance (ETS) database.*<br>• *To assess patient satisfaction of treatment received in primary care compared with secondary care.*<br>• *To evaluate cost effectiveness of LTBI treatment in primary care compared with treatment in secondary care.*<br><br>***1.3. Additional objectives***<br>• *To identify factors associated with non-*

	acceptance, non-adherence or poor completion of treatment.<br>• *To develop a research infrastructure to form the basis of further research proposals within Barts Health and in collaboration with other UCLP hospitals*
**Number of Participants/Patients**	*We will randomise at least 20 GP surgeries and recruit 1014 patients. 507 will be treated in primary care and 507 patients referred to secondary care. The sample size required will fall if additional GP surgeries are randomised.*
**Main Inclusion Criteria**	*Eligible patients will be*<br>• *LTBI-positive,*<br>• *aged 16-35*<br>• *have entered the UK less than 10 years ago from a country with a TB incidence of greater than 150/100,000, or sub-Saharan Africa.*
**Statistical Methodology and Analysis (if applicable)**	*Comparison of treatment between the two strategies will be performed by fitting a multilevel mixed effect logistic regression model with treatment completion as the dependent variable, strategy as a fixed effect factor, and a random effect for each cluster (GP practice). In a secondary analysis, this model will include potential confounders recorded at patient level and cluster level. Potential confounders will include participants' age, ethnicity, TB risk factors and other patient characteristics believed to be prognostic, and GP practice characteristics including size of practise, number of GP per practise and number of migrants registering with practice per annum. An objective strategy for selection of potential confounders to adjust for will be specified before analysis begins. Similar analysis will be undertaken for each secondary outcome measure including acceptability of treatment and adverse effects.*
**Proposed Start Date**	01.04.2016
**Proposed End Date**	31.12.2019
**Study Duration**	***36 months***

4. INTRODUCTION

4.1 Background

One-third of the world's population is estimated to have latent tuberculosis infection (LTBI) and there is a lifetime risk of progression to active tuberculosis (TB) in 5-10% of immunocompetent persons. [1] In the UK rates of active TB have steadily risen over the last 20 years and the UK has now the second highest TB rate in Western Europe. [2] Barts Health NHS trust has the highest number of notifications of TB compared to all other Trusts in England and manages 10% of all active TB cases in the UK. Most active TB cases in London occur in migrants from countries with a high TB prevalence such as the Indian subcontinent and sub-Saharan Africa who have acquired LTBI outside the UK and reactivate in the first 5-10 years after arrival. [3] There is substantial evidence that risk of progression from LTBI to active TB is reduced by treating LTBI. [4] Patients who do not complete treatment are known to have a higher risk of developing active disease than those who complete treatment (unpublished data, [5])

Screening with a blood test – the Interferon-gamma release assay (IGRA) - has revolutionised diagnosis of LTBI. These assays measure interferon-gamma release when mycobacterial antigens evoke a strong and specific T-helper 1 type cell-mediated immune response and measure response to region of difference 1 (RD1) specific antigens resulting in less cross-reactivity with non-tuberculous mycobacteria and no cross reactivity with Bacille Camille Guerin (BCG) vaccination compared to tuberculin skin test (TST) [6]. Uptake of screening, however, and adherence to LTBI treatment is poor in hospital based TB clinics. [7] We pioneered TB screening in general practice: our locally conducted trial of an educational outreach intervention to promote screening for active and latent TB in East London general practices showed that screening is feasible in primary care and detects a high proportion of latent and active TB cases.[8] Virtually all Hackney practices took up TB screening after this trial was completed. Nevertheless, Hackney patients were treated in secondary care. Universal screening and treatment of all eligible migrants in a hospital setting will almost certainly be more expensive than treatment in primary care and currently overstretches the limited capacity of hospital TB services and commissioning budgets.

Secondary care referral for LTBI treatment leads to significant numbers of non-attendance in TB clinics [9] as the ongoing PREDICT latent TB prognostic cohort study has shown (personal communication I Abubakar). Acceptability of and adherence to treatment in migrants with LTBI is thought to be low partly because individuals screened do not have symptoms and perceive the risk of developing active TB as low. [7] Factors for low treatment uptake and adherence have not been evaluated sufficiently and strategies to improve LTBI treatment adherence such as peer support [10], financial incentives [11] and directly observed therapy (DOT) have been mainly evaluated in secondary care by using INH or Rifampicin monotherapy.[12] Home visits [13] or mobile health clinics [14] to monitor LTBI treatment have shown to improve completion rates but these strategies are expensive and cannot be employed in a large scale LTBI treatment project. A

secondary care based pharmacist-managed clinic for treatment of LTBI in health care workers has shown very high completion rates.[15] But there is no evidence of uptake and treatment completion for currently used shorter regimens (3 months Rifampicin/INH combination therapy) in a primary care setting. Many migrants are asylum seekers or refugees who often do not have NHS numbers and are more likely to attend an appointment with their GP than a hospital TB clinic because of fear having to pay for treatment.[16] Patients prefer to be seen in primary care due to reduction in waiting times [17], having flexibility of appointment times [18] and it is therefore probable that migrants are more likely to attend appointments in primary care than secondary care. The risk of adverse effects especially drug induced liver injury (DILI) using Rifampicin and INH in combination is low when administered in patients below the age of 35 and in individuals without underlying liver disease such as hepatitis B and C [19, 20](unpublished data) and adverse effects do not seem to have a major impact on patients' decisions to discontinue treatment before completion.[21]

There is a strong desire both locally and nationally, to support quality improvement in primary care and to shift care from hospitals to community settings closer to patients' homes. Our trial takes advantage of a unique opportunity - the recently initiated migrant LTBI screening and treatment project, commissioned as an Extended Primary Care service by Newham CCG in view of the extremely high rates of active TB occurring mainly in young migrants from high TB incidence countries.

The trial will compare LTBI treatment adherence, acceptance, safety and cost effectiveness among eligible migrants in primary care compared to secondary care. GP practices will be randomised to refer patients to secondary care (control group) or to provide treatment in primary care. The trial will support an education and training programme focussing on treatment of LTBI for all GPs, health care assistants and community pharmacists in Newham who are delivering this service. For the first time this study will be able to provide robust evidence on the most acceptable and cost-effective setting for LTBI treatment.

4.2 Current Extended LTBI Service in Newham

The LTBI screening and treatment service was initiated in July 2014 in primary care in Newham.

In the extended service, any patient who registers with a GP practice and is under 35 years of age, has lived in the UK less than 10 years and is from a country with an incidence of TB greater than 150 per 100 000, or sub-Saharan Africa, is offered screening for LTBI. GPs may also invite patients already registered with their practices for screening if they meet the criteria listed above.

The initial testing includes an IGRA, a Full blood count (FBC), urea and electrolytes (U&E), liver function tests (LFT), C-reactive protein (CRP), Human Immunodeficiency Virus (HIV) serology and Hepatitis B and C viral serology. If the IGRA is positive the GP is informed and reviews the patient to exclude active TB. If they are asymptomatic, they are then offered a chest x-ray (CXR). If their CXR is normal and the blood tests: LFTs, CRP, and viral serology are normal (based on the local service specification), the GP will offer the patient treatment for LTBI. An electronic prescription is sent to a community pharmacist, who has been trained as part of the

extended service to provide LTBI treatment. They co-ordinate treatment, including arranging a set of LFTs at two weeks after treatment has been started.

As part of the extended service patients are asked to complete questionnaires about knowledge related to LTBI/TB, adherence and adverse effects from treatment and the Medication Adherence Report Scale (MARS-5). They are asked to complete a patient satisfaction questionnaire at the end of treatment. The design of this clinical trial is based on the the current Newham extended LTBI service specification to allow for a comparison of this model of care with treatment in secondary care.

From an evaluation of the first year of the project based on data extracted from EMIS web and from pharmacy records, 7947 patients were offered screening in Newham. Of these, 2982 had an IGRA test and 901 patients were found to be IGRA positive. 337 patients have completed treatment for LTBI in community during this period. We have obtained positive feedback from GPs and community pharmacists regarding the service. Most patients treated in primary care had minor adverse effects. 11 patients were diagnosed with active TB having been picked up during screening for LTBI.

5. TRIAL OBJECTIVES

5. 1. Primary objective

- To determine whether an innovative programme of treating latent tuberculosis infection (LTBI) in primary care increases LTBI treatment completion (defined as taking at least 90% of antibiotic dosages based on pill count) compared with current practice (treatment in secondary care).

5.2. Secondary objectives

- To assess the proportion of patients completing greater than 80% and 85% of antibiotic dosages based on pill count.
- To assess the proportion of patients who are adherent to treatment based on the MARS5 tool, prescription collection and point-of-care urine testing for metabolites of Isoniazid.
- To describe the proportion of individuals in the two treatment arms who accept LTBI treatment.
- To assess the incidence of adverse effects of treatment for LTBI including drug induced liver injury (DILI) in both arms.
- To assess rates of active tuberculosis (TB) cases in primary and secondary care during and after the study period measured as case notifications to Public Health England (PHE) using the Enhanced TB surveillance (ETS) database.
- To assess patient satisfaction of treatment received in primary care compared with secondary care.
- To evaluate cost effectiveness of LTBI treatment in primary care compared with treatment in secondary care.

5.3. Additional objectives

- To identify factors associated with non-acceptance, non-adherence or poor completion of treatment.
- To develop a research infrastructure to form the basis of further research proposals within Barts Health and in collaboration with other UCLP hospitals (nearly a third of the national TB burden).

6. METHODOLOGY

6.1 General Methodology

6.11 Practice recruitment

To ensure that small and large practices from the whole borough of Newham are equally represented in both arms, we will stratify practises according to size and number of migrants with LTBI identified since the extended service was rolled out in primary care in July 2014. We will provide a financial incentive to GP Surgeries participating in the study. Community Pharmacists will be given an incentive per patient treated.

6.12 Practice randomisation

GP practices willing to participate in the trial will be randomised with allocation concealment, by a statistician external to the trial, to the intervention arm (treatment in primary care) or control (referral to secondary care). Before randomisation, data from the practices to be randomised will be collected relating particularly to practice size, migrants screened and treated for LTBI. Once these data have been collected and examined, to avoid imbalance, randomisation will be conducted either by simple permutation within two strata (defined by the practice data) or by restricted randomisation.

6.13 Consent

All patients will be given a Patient Information Sheet that explains the study and what participation will entail.

The study has two separate areas where the issue of consent arises. The first relates to consent for participation in the trial, and for this the study will assume valid implied consent. The second relates to consent for data collection, and for this participants will be asked for verbal consent that will be documented within the medical records. The reasons for these approaches are outlined in detail later in the protocol.

6.14 Data collection

Basic data on age, ethnicity, socioeconomic status, substance misuse and pre-existing medical conditions (such as diabetes) will be recorded in all patients electronically in primary care using EMIS. Information on adherence and adverse effects will be obtained every month and recorded electronically by Community Pharmacists and in TB Clinic, according to existing protocols (see section 4.2 and 7.4)

6.2 Inclusion Criteria

Patients with LTBI aged 16-35 and who have entered the UK less than 10 years ago from a country with a TB incidence of greater than 150/100,000, or sub-Saharan Africa.

In the protocol, LTBI is defined as a positive IGRA test without any symptoms or physical signs of active TB and no evidence of active TB on Chest X-ray.

6.3 Exclusion Criteria

Patients who do not meet the criteria for the standard Newham primary care treatment protocol will be excluded which include:

1) Pregnant or breastfeeding women.
2) Patients requiring medications that cannot be safely taken with Rifinah
3) HIV infection.
4) Individuals with known liver disease, or abnormal liver function tests (LFTs) as defined in the Newham liver function protocol.
5) Diagnosis of cirrhosis (jaundice, hematemesis, ascites or previous episodes of liver encephalopathy).
6) Chronic or active hepatitis B or hepatitis C virus infection.
7) Previous treatment for TB or LTBI.
8) Individuals who are unable to consent or who would usually be offered LTBI treatment under DOT because of their mental or social disabilities or those with drug or alcohol abuse.
9) Evidence of active TB (based on history, examination, blood tests and/or Chest X-ray finding).

6.4 Data Collection and Follow up for Withdrawing Participants

A participant can be withdrawn from the trial treatment if, in the opinion of the investigator or the care providing clinician or clinical team, it is medically necessary to do so. Withdrawal from follow-up is the decision of the participant. However, withdrawn participants can bias clinical trial results and reduce the power of the trial to detect important differences. With any post randomisation exclusions, the study personnel will make every effort to obtain, and record, information about the reasons for violation, any adverse events and to follow-up the patients for all safety and efficacy outcomes, as appropriate. If a patient decides after referral to secondary care or when treated in primary care that he/she does not wish to participate any further in the LTBI trial, he/she may withdraw him/ herself from the trial. We will aim to document the reason for self-withdrawal. Clear distinction will be made as to whether the participant is withdrawing from trial whilst allowing further follow-up, or whether the participant refuses any follow-up. If a participant explicitly withdraws consent to have any further data recorded their decision will be respected and recorded on the final study form. All communication surrounding the withdrawal will be noted in the study records and no further data will be collected for that participant. The patient information sheet explains that patients may withdraw from the study at any point and that no further data will be collected.

6.5 Plan of investigation and Implementation

6.51 Study conduct/Study design

Prospective cluster randomised trial of LTBI treatment in primary care based at GP practices who are part of the Newham TB screening and treatment project and who have agreed to take part in the trial. The practises will be randomised with allocation concealment by a statistician external to the trial, either to treat all their IGRA positive patients in primary care (intervention) or refer them for treatment in secondary care (standard treatment). Migrants, who are eligible for screening under the extended service (aged 16-35 and from a country with TB incidence greater than 150/100,000, or sub-Saharan Africa [22] who have been in the UK for less than 10 years and do not have evidence of active TB) will be eligible for trial enrolment.

Figure 3 - Study Scheme Diagram

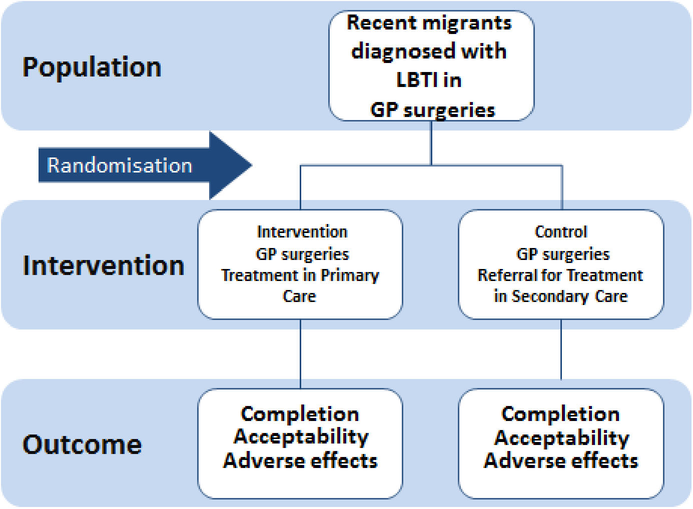

6.52 Setting

GP practices in Newham and the TB clinic Shrewsbury Road (Newham Hospital)

7. STUDY PROCEDURES

7.1 Consent Procedures

All issues of consent have been developed with co-applicant Professor Richard Ashcroft, Professor of Bioethics, Queen Mary University of London.

All eligible patients will be given a Patient Information Sheet (appended) that explains the reasons for the trial and outlines what participation will entail. This will be

available in the main languages spoken locally. The study has two separate areas where the issue of consent arises. These are the consent for trial participation and the consent for data sharing.

The study will assume valid implied consent for participation in the trial. This design has been used in two other recent trials related to TB and HIV screening [8,27]. The reasons for this approach are outlined below:

Firstly, the study compares two methods of care that can both be considered "standard routine care". In Newham, the current Extended LTBI Screening and Treatment Programme manages patients with LTBI in the community. In the rest of the UK, patients identified through the screening for LTBI are referred to secondary care for treatment. Whilst there is genuine clinical equipoise as to whether patients treated for LTBI in the community are more likely to complete treatment than those treated in secondary care, there are no specific risks to the patient from being allocated to either arm of the study. Furthermore, participating in the trial does not require the patient to undertake any additional blood tests or radiological investigations other than what would is required as part of the routine care of any patient with LTBI. Thus, the intervention is without any specific risks.

Secondly, the study randomises practices rather than patients therefore a registering patient does not face an option of which group they are in, this has already been allocated by practice randomisation.

Thirdly, as screening occurs at the point of registration to a GP practice, an unpredictable event, it is not feasible to have a member of the research team present to consent each patient. The alternative, that doctors and practice nurses consent patients is also impractical. It would present an unworkable burden for these staff.

The Patient Information Sheet will explain very clearly that patients can opt out of the trial at any point without any impact on their care.

The study will ask for verbal consent from patients to allow the research team to access their data.

In the current Extended LTBI Screening and Treatment Programme in Newham, patients are asked for verbal consent for their data to be shared with Public Health England (PHE). This is recorded electronically.

It is not feasible to take formal written consent for data collection for the same reasons it is not feasible to collect written consent for participation. The study will adopt the same approach as PHE and obtain verbal consent for data sharing at the point the patient is seen by a doctor. This will be recorded in the notes.

The Patient Information Sheet (PIS) will explain clearly how patient data is collected and how they can opt out of the study.

Ineligible participants

Queen Mary University of London

Patients who are ineligible for the trial, or who do not wish to participate in the trial will be managed outside the trial according to usual practice.

7.2 Screening, Enrolment

7.21 New patients
Participating GP Practices will offer LTBI screening to all patients when they register with the practice if they meet the pre-defined eligibility criteria: aged 16-35 and entered the UK less than 10 years ago and were born in a country with a TB incidence of greater than 150/100,000, or sub-Saharan Africa. At this point, patients will be given the Patient Information Sheet (PIS) for the trial.

7.22 Existing patients
Existing eligible patients who are already registered with a GP and have either never been offered or have not declined screening previously may be given a Patient Information Sheet (PIS) and a blood form for an IGRA test.

Eligible patients who are already registered and who had a positive IGRA test prior to the trial opening for recruitment, and have not had a subsequent follow up appointment, may be given a PIS and invited to attend the GP for counselling and the offer of treatment.

Eligible patients who are already registered and who had a positive IGRA result prior to the trial opening for recruitment, and have had a subsequent follow up appointment but no prescription for treatment issued, may be given a PIS and invited to a further appointment for counselling and the offer of treatment. This group will be treated as a separate cohort and analysed separately from the main trial.

7.3 Randomisation Procedures

GP practices in the trial will be randomised with allocation concealment, by a statistician external to the trial, to the intervention arm (treatment in primary care) or control (referral to secondary care). Before randomisation, data from the practices to be randomised will be collected relating particularly to practice size, migrants screened and treated for LTBI. Once these data have been collected and examined, to avoid imbalance, randomisation will be conducted either by simple permutation within two strata (defined by the practice data) or by restricted randomisation.

7.4 Planned intervention

7.41 Trial Intervention

The trial intervention is the setting of LTBI Treatment: either in the community, managed by the GP and community pharmacist or in secondary care, managed by the TB doctor and nurse.

All eligible patients (those aged 16-35 and who have entered the UK less than 10 years ago from a country with a TB incidence of greater than 150/100,000, or sub-

Saharan Africa) are offered screening for LTBI in the form of an IGRA and other blood tests (see section: Current Extended LTBI service in Newham) when they register to join a GP practice as per routine care within Newham. All patients eligible for screening will be given a Patient Information Sheet explaining the study.

All IGRA positive patients who fulfil the inclusion criteria (see section 6.3) are eligible to enter the study. Those with a positive IGRA who are not eligible for treatment in primary care will be referred to secondary care.

7.42 The Intervention: Managing LTBI in primary care

Patients with a positive IGRA not meeting any of the exclusion criteria will be invited to see their GP. At this review, the GP will take a brief history and perform a physical examination, to exclude a diagnosis of active TB. The patient will also be asked to complete a questionnaire about their understanding of LTBI. If there is no evidence of active TB, the GP will explain to the patient the diagnosis of LTBI, and offer the patient treatment pending a Chest X-ray (CXR). The patient will then attend local radiology services for the the CXR, and the GP will be sent the result. If the CXR shows no signs of active TB, the GP will generate an electronic prescription that can be collected from community pharmacies participating in the trial. The patient will be asked to attend a listed pharmacy to start treatment.

Routine Treatment for LTBI is shown in the table below:

**Adult patients <50kg**	**Adult patient >50kg**
Rifinah 150, 3 tablets daily<br>(Total dose: Isoniazid 300mg, Rifampacin 450mg daily)<br>Pyridoxine 25mg once daily	Rifinah 300, 2 tablets daily<br>(Total dose: Isoniazid 300mg, Rifampacin 600mg daily)<br>Pyridoxine 25mg once daily
**Duration: 3 months**	

At the initial visit, the community pharmacist will explain to the patient the treatment including advice about adverse effects. They will explain that the patient must attend for repeat liver function tests (LFTs) after 2 weeks, and give the patient the form to obtain this. The patient will be issued with a 1-month supply of medication.

In case of adverse events and drug induced liver injury (DILI), GPs and community pharmacists will follow the existing Newham treatment manual and standardised referral pathway to secondary care.

The pharmacist will contact the GP after the LFTs have been performed to check the results. If the patient has failed to attend for the test performed they will be contacted by the pharmacist and reminded to attend for the test.
If a patient has failed to attend for a blood test at the point that they present to collect the second month of treatment the pharmacist will do a symptom and adherence check. If the patient does not any report side effects and is well, the pharmacist will

issue the second months supply of treatment. The pharmacist will remind the patient that they should attend for the LFT check and will continue to check for the result. The pharmacist will advise the GP if there is no evidence of LFT results after two reminders to the patient.

If the patient has normal LFTs when they attend to collect their second month's prescription, the pharmacist will ask about adverse effects and assess adherence. They will then issue the patient with a further months supply of medication.

Adherence is assessed using the Medication Adherence Report Scale (MARS5), a pill count and a point-of-care urine test for metabolites of Isoniazid (IsoScreen).
At the third month, the pharmacist will again ask about adverse effects and assess adherence using the same approach as previously. The patient will also be asked to complete a patient satisfaction questionnaire.
At the end of treatment, the patient will be asked to return to the pharmacist for review to discuss any adverse effects and assess adherence (MARS5/pill count/Urine point-of-care). If the patient fails to attend they will be contacted by telephone. If contact with the patient has not been made by the pharmacist, the research team will contact the patient to request review to obtain a pill count, if this is not possible, information will be obtained by patient self-report, which will supersede pill count.

7.43 The Control: Managing LTBI in secondary care

In the control arm, patients with a positive IGRA not meeting any of the exclusion criteria will be informed that they are being referred to secondary care (TB Clinic) for review.

At this review, the TB doctor will take a brief history, perform a physical examination, and organise a CXR to exclude a diagnosis of active TB. If there is no evidence of active TB, the TB doctor will explain the diagnosis of LTBI, and offer the patient treatment. The patient will also be asked to complete a brief questionnaire about their knowledge of LTBI.

Treatment is identical to the intervention arm.

The TB nurse will explain to the patient about the treatment including advice about adverse effects. The patient will be given a 1-month supply of medication and asked to attend for repeat LFTs in 2 weeks. The TB Nurse will check the LFT results. If the patient has failed to attend for the test performed they will be contacted and reminded to attend for the test.

If a patient has failed to attend for a blood test at the point that they present to collect the second month of treatment the TB nurse will do a symptom and adherence check. If there are no concerns, the TB nurse will issue the second months treatment and will advise the patient to attend for LFT check. The result will be followed up and the TB doctor advised if the patient continues to fail to attend for LFT.

If the patient has normal LFTs when they attend to collect their second month's prescription, the TB nurse will ask about adverse effects and assess adherence. They will then issue the patient with a further months supply of medication.

Adherence is assessed using the Medication Adherence Report Scale (MARS5), a pill count and a urine sample to perform a point-of-care test for metabolites of Isoniazid (IsoScreen).

At the third month, the TB nurse will again ask about adverse effects and assess adherence using the same approach as previously. They will also be asked to complete a patient satisfaction questionnaire.

At the end of treatment, the patient will be invited to attend to see the TB nurse for review to discuss any adverse effects and assess adherence (MARS5/pill count/Urine point-of-care). If the patient fails to attend they will be contacted by telephone, if a face-to-face review cannot be arranged, information will be obtained by patient self-report.

7.5 Data collection Procedure

Data will be collected during routine care by the patient's usual health care team. Data will be entered in GP Surgeries using EMIS web by a Health Care Assistant at Registration and by the GP during consultations. In the primary care arm of the trial, data will be collected electronically by Community Pharmacists using a purpose built web-based system (Webstar Health). This will include information from questionnaires, the MARS5 tool, pill counts and the results of urine point-of-care tests. In secondary care, data will be collected electronically. Data will be extracted from EMIS, Webstar and secondary care and combined to form a single database. This will be pseudoanonimised prior to analysis by the trial statistician.

7.6 Proposed outcome measures

7.61 Primary Outcome –Treatment completion

All IGRA positive patients who complete 90% of prescribed therapy as assessed by pill count. Pill counts will be recorded monthly, where a patient fails to bring their medication information will be obtained by patient report. Treatment completion will be assessed at the end of treatment by the community pharmacist in primary care and the TB nurse in secondary care. In order to avoid bias, an independent blinded researcher will verify treatment completion where there is uncertainty.

7.62 Secondary Outcomes

- Treatment completion rates of greater than 80% and 85% of antibiotic dosages based on pill count will also be assessed.
- Treatment Adherence will be assessed using the five-point adherence questionnaire (MARS5 tool), prescription collection and in cases of uncertainty a point-of-care urine testing for metabolites of Isoniazid (Isoscreen) at monthly intervals.
- Treatment acceptance will be assessed by calculating the proportion of eligible patients that accept therapy; this is defined as those initiating

treatment and attending TB clinics and community pharmacies on at least one occasion.

- The number of patients on treatment having adverse events including DILI leading to discontinuation of treatment or hospitalisation. Safety of treatment will be assessed every month by the pharmacist in primary care and by the TB nurse in secondary care.
- The incidence of active TB occurring within 2 years after enrolment. TB incidence in the intervention and control group will be compared and there will be a sub-analysis of examining those who did or did not accept or complete treatment. This will be performed through matching the study population with the national Enhanced TB Surveillance System, where information on all reported TB cases nationally are recorded.
- Assessment of patient satisfaction using a standardised treatment satisfaction questionnaire at the end of treatment. (Appendix 2)
- Evaluation of cost-effectiveness of treatment in primary care compared to secondary care.

7.63 Additional outcomes

- Identification of factors associated with treatment non-acceptance by using a standardised questionnaire to investigate patients' understanding and knowledge of LTBI; and exploring risk factors and barriers to LTBI treatment.
- Identification of factors associated with treatment non-adherence by using a standardised questionnaire at the end of treatment investigating causes of non-adherence.

7.7 Study implementation

Adherence to treatment will be monitored using pill counts and a five-point adherence questionnaire (MARS-5 tool) and a urine sample for a point-of-care test for metabolites of Isoniazid (Isoscreen). Each month patients will be asked to complete a questionnaire regarding adverse effects to treatment. All participants will be asked to complete a patient satisfaction questionnaire regarding care. Translation of questionnaires and validation of the translation will be done according to existing protocols to assure accuracy in interpretation and analysis.

Table 1: Outlining schedule of interventions for each participant.

	Day 0	Day 15	Day 30	Day 60	Day 90
Questionnaire (Understanding and knowledge of LTBI)	X				
Blood tests for LFTs		X			
Mars- 5 tool questionnaire / pill count			X	X	X
Questionnaire (adherence/ adverse effects)			X	X	X
Urine test (Colour and INH metabolites)[1]			X	X	
Patient satisfaction questionnaire				X	

1 A minimum of two urine tests should be performed during the three months of treatment. These should be done at the review visit for months 1 and 2 of treatment, although the second may be done at the final visit instead if the patient was unable to provide a sample at either of the earlier visits, or additionally at the final visit if there are concerns about adherence,

7.8 Schedule of intervention for each visit:

Every patient will be asked to complete an adherence questionnaire (MARS 5 Tool) at each pharmacy visit in primary care and each clinic visit in secondary care.

7.9 End of Study Definition

When the last enrolled participant has completed treatment, the REC will be notified of the trial completion. The final study report will be completed within 24 months after the trial completion.

8. STATISTICAL CONSIDERATIONS

8.1 Sample size

From published data and our retrospective review, 70% of patients currently complete LTBI treatment.[19, 26] We expect treatment completion in primary care to improve by 15% compared with secondary care (from 70% to 85%). To detect this difference with 80% power, and 5% significance level, an individually randomised trial

would require 268 participants. We shall conduct our trial in a minimum of 20 GP practices (10 intervention and 10 control). With 20 practices we would need to adjust for the effect of clustering by increasing the sample size to 780 participants (or 39 patients per practice). To allow for loss to follow up and treatment non-acceptance we would inflate the required sample size by 30% to 1014 participants (51 per GP practice).

As the number of GP Practices increases the required sample size falls. For example, if the trial is conducted in 24 practices, the sample size required falls to 740, allowing for the expected loss to follow up as described above. This calculation has assumed equal numbers of participants in both groups, an ICC of 0.05 (Griffiths Lancet 2007) [8] and a design effect of 2.9.

8.2 Recruitment

The large number of eligible participants and practices will allow successful recruitment to this study. Each GP practice in Newham newly registers on average 260 new migrants per annum who fulfil our eligibility criteria (Data from the Barts Clinical Effectiveness Group). In addition, an average of 278 eligible patients would already be registered in each GP practice in Newham. As a result of the current LTBI screening pilot community and awareness activities by “Healthwatch” and other charities, we assume that 70% of these patients will agree to be screened and of these 25 % will be IGRA positive (based on findings of the PREDICT study). Even if the trial is conducted in the minimum number of 20 GP practices, within the anticipated running period of 24 months we expect that a total of 7,800 patients would register across the practices in addition to the 5,560 patients who are already registered. Of these, we would expect 9,352 patients to accept screening and 2338 to be IGRA positive, more than twice the required sample size.

8.3 Analysis

Comparison of treatment between the two strategies will be performed by fitting a multilevel mixed effect logistic regression model with treatment completion as the dependent variable, strategy as a fixed effect factor, and a random effect for each cluster (GP practice). In a secondary analysis, this model will include potential confounders recorded at patient level and cluster level. Potential confounders would include participants’ age, ethnicity, TB risk factors and other patient characteristics believed to be prognostic, and GP practice characteristics. An objective strategy for selection of potential confounders to adjust for will be specified before analysis begins. Similar analysis will be undertaken for each secondary outcome measure including acceptability of treatment and adverse effects.

9. ETHICS

This protocol and any subsequent amendments, along with any accompanying material provided to the participant in addition to any advertising material will be submitted by the Investigator to an Independent Research Ethics Committee. Written Approval from the Committee will be obtained and subsequently submitted to the

JRMO to obtain Final R&D approval. The trial can only start after approval from a Research Ethics Committee and the local R&D “Sign-off” from Barts Health. If there is any further safety information which may result in significant changes in the risk/benefit analysis, the Patient Information Sheet (PIS) will be amended accordingly and submitted to REC for revision and approval.

Patients are free to withdrawal at any point of the study. If a patient withdraws during the study all data collected up to point of withdrawal shall be retained unless the patient requests otherwise at the point of withdrawal.

10. DATA HANDLING AND RECORD KEEPING:

10.1 Confidentiality

The Investigator will ensure that patient anonymity is protected and maintained. They will also ensure that patient identities are protected from any unauthorised parties. Information with regards to study patients will be kept confidential and managed in accordance with the Data Protection Act, NHS Caldicott Guardian, The Research Governance Framework for Health and Social Care and Research Ethics Committee Approval.

The trial will collect personal data and information about the participants either directly or from their clinical team. The data will be entered onto a secure computer database, either by the research team or directly via a secure internet connection. Any data processed by those outside the research team (research registrar, nurse or project co-ordinator) will be anonymised. All personal information obtained for the trial will be held securely and treated as (strictly) confidential. All staff share the same duty of care to prevent unauthorised disclosure of personal information. No data that could be used to identify an individual will be published

The Chief Investigator, Dr. Heinke Kunst is the ‘Custodian’ of the data.

10.2 Required Study Documents

- A signed protocol and any subsequent amendments
- Current and Superseded Patient Information Sheets
- Current and Superseded GP letters
- Current and Superseded Posters
- Current and Superseded CRFs
- Indemnity documentation from sponsor
- Conditions of Sponsorship from sponsor
- Conditional/Final R&D Approval
- Signed site agreements
- Ethics submissions/approvals/correspondence
- CVs and GCP certificates of CI and site staff
- Laboratory accreditation letter, certification and normal ranges for all laboratories to be utilised in the study
- Delegation log

- Staff training log
- Identification log
- Enrolment log
- Monitoring visit log
- Correspondence relating to the trial
- SAE reporting plan for the study

10.3 Case Report Forms

Data will be entered by the direct health care team (GP, community pharmacist, TB doctor or TB nurse). In primary care, this will be entered electronically (EMIS and Webstar Health). In secondary care this will also be entered electronically on to a secure database. Data will then be extracted and combined into a single database.

Suitably qualified members of the study team, as documented on the trial delegation log will be responsible for the completion of the database.

10.4 Data collection, processing and monitoring

All trial data will be managed according to the CEG data management SOP's.
Data will be;

- Entered directly onto the database where possible (paper CRFs will be used as a backup if required)
- Screened for out-of-range data, with cross-checks for conflicting data within and between CRF using computerised logic checking screens
- Referred back to the relevant centre for clarification in the event of missing items or uncertainty

Paper CRFs (if needed) will be verified and processed on site by trial coordinators or other delegated members of the study team for data entry to the trial database.

10.5 Central statistical monitoring

All data will be monitored centrally (at the CEG) for consistency, viability and quality using bespoke data management systems. Central statistical monitoring by UCL will examine patterns of recruitment, characteristics of patients, date of recruitment, etc. The trial programmer will run trial-specific programs to extract certain fields from the database (as requested by the Chief Investigator or Trial Statistician) and to cross-check specific information. These fields may include measures of eligibility criteria and management after trial entry. The trial programmer and Chief Investigator will review the results generated for logic and for any patterns or problems. Outlier data will be investigated. The Chief Investigator and Trial Statistician will decide if any action is required.

10.6 Record Retention and Archiving

During the course of research, all records are the responsibility of the Chief Investigator and must be kept in secure conditions. When the trial is complete, it is a requirement of the Research Governance Framework and Trust Policy that the records are kept for a further 20 years. For trials involving Barts Health Trust patients, undertaken by Trust staff, or sponsored by Barts Health Trust or QMUL, the approved repository for long-term storage of local records is the Trust Modern Records Centre, which is based at 9 Prescot Street.

11. LABORATORIES

All blood tests in primary care will be performed by laboratories used in primary care and reports of these tests will be sent electronically to the TB clinic when patients are referred to secondary care.

All blood tests performed in secondary care are performed by Barts Health laboratories.

11.1 Sample Collection/Labelling/Logging

Sample collection requirements will be performed according to the existing Newham service. NHS Samples will be collected, labelled and processed according to NHS standard practice and logged onto the NHS database.

11.2 Sample Receipt/Chain of Custody/Accountability

Samples will be checked by laboratory staff as per NHS standard practice prior to processing. Any inconsistencies will be referred back to the person collecting the sample or the research team. All samples received and processed will be logged onto the NHS database.

11.3 Sample Analysis Procedures

Sample analysis will be conducted according to the NHS standard operating procedures.

11.4 Radiology

All patients with a positive IGRA test will have a chest radiograph performed to exclude active TB according to existing NHS protocols which will be reported by the Newham radiology team. If a CXR shows evidence of active TB, the TB clinic will be informed electronically so that patients can be reviewed in the next available TB clinic

11.5 Data Recording/Reporting

Pathology reports from of laboratory results will be recorded on EMIS in primary care and in secondary care lab reports will be printed and filed in the medical records as per usual NHS practice. All path reports will be transcribed to the trial CRF/database

by a delegated member of the trial team in secondary care. The CRF will be pseudonymised with all participant identifiers removed.

12. TOOLS

12.1 Questionnaires/Pill counting/MARS5 Tool

A Questionnaire will be used to assess the patients' knowledge of LTBI prior to starting treatment. In the primary care arm of the trial, the answers will be recorded electronically by the GP or practice nurse. In secondary care, the answers will be recorded electronically by the TB doctor or TB Nurse. These questionnaires have been piloted with patients at a GP practice within Newham and updated based on their feedback.

The community pharmacists and TB nurses will assess patients' adherence and completion of treatment by using pill counting and collection of monthly prescriptions. In addition, The Medication Adherence Report Scale (MARS-5) questionnaire and a urine dipstick to check for INH metabolites will be used to complement this data.

The MARS5 tool has been included in the Newham service specification to be used routinely in primary care. It has also been introduced for routine use in secondary care at in the TB clinic at Newham Hospital. The data will be entered electronically into a web based pharmacy database according to existing protocols in primary care. In secondary care, adherence and completion of treatment data will be entered into the patients' clinical notes and transferred to electronic CRFs. Two independent investigators blinded to the setting will determine treatment completion in individuals where treatment completion is unclear. The community pharmacists in primary care and TB nurses in secondary care complete questionnaires about adverse events according to existing protocols. These paper records will be transferred into CRFs. The patients are asked to complete a patient satisfaction questionnaire that will be transferred into patients' CRFs.

13. SAFETY REPORTING

13.1 Adverse Events (AE)

An AE is any untoward medical occurrence in a subject to whom a medicinal product has been administered, including occurrences which are not necessarily caused by or related to that product. An AE can therefore be any unfavourable and unintended sign (including an abnormal laboratory finding), symptom or disease temporarily associated with study activities.

Notification and reporting Adverse Events or Reactions

If the AE is not defined as SERIOUS, the AE is recorded in the study file and the participant is followed up by the research team. The AE is documented in the participants' medical notes (where appropriate) and the CRF.

13.2 Serious Adverse Event (SAE)

A serious adverse event (SAE) is defined as an untoward occurrence that:

(a) results in death;
(b) is life-threatening;
(c) requires hospitalisation or prolongation of existing hospitalisation;
(d) results in persistent or significant disability or incapacity;
(e) consists of severe hepatic failure
(f) is otherwise considered medically significant by the investigator.

An SAE occurring to a research participant should be reported to the main REC where in the opinion of the Chief Investigator the event was:

- Related – that is, it resulted from administration of any of the research procedures, and
- Unexpected – that is, the type of event is not listed in the protocol as an expected occurrence.

Notification and Reporting of Serious Adverse Events

Serious Adverse Event (SAEs) that are considered to be 'related' and 'unexpected' are to be reported to the sponsor within 24 hours of learning of the event and to the Main REC within 15 days in line with the required timeframe. For further guidance on this matter, please refer to NRES website and JRMO SOPs

13.3 Urgent Safety Measures

The CI may take urgent safety measures to ensure the safety and protection of the clinical trial subjects from any immediate hazard to their health and safety. The measures should be taken immediately. In this instance, the approval of the REC prior to implementing these safety measures is not required. However, it is the responsibility of the CI to inform the sponsor and Main Research Ethics Committee (via telephone) of this event immediately.

The CI has an obligation to inform both the Main REC in writing within 3 days, in the form of a substantial amendment. The sponsor (Joint Research Management Office [JRMO]) must be sent a copy of the correspondence with regards to this matter. For further guidance on this matter, please refer to NRES website and JRMO SOPs.

13.4 Annual Safety Reporting

The CI will send the Annual Progress Report to the main REC using the NRES template (the anniversary date is the date on the MREC "favourable opinion" letter from the MREC) and to the sponsor. Please see NRES website and JRMO SOP for further information

13.5 Overview of the Safety Reporting responsibilities

The CI has the overall pharmacovigilance oversight responsibility. The CI has a duty to ensure that pharmacovigilance safety monitoring and reporting is conducted in accordance with the sponsor's requirements. Each participating GP surgery will be responsible for reporting all SAEs to the chief investigator immediately so that a decision can be made as to whether this needs to be reported to the sponsor, QA manager and MREC. The CI will keep a log of all SAE's reported by the participating for reporting to the REC and Trial Steering Commitee.

14. MONITORING &AUDITING

14.1 Audit and Inspection

Auditing: Definition "A systematic and independent examination of trial related activities and documents to determine whether the evaluated trial related activities were conducted, and the data were recorded, analysed and accurately reported according to the protocol, sponsor's standard operating procedures (SOPs), Good Clinical Practice (GCP), and the applicable regulatory requirement(s)."

A study may be identified for audit by any method listed below:
1. A project may be identified via the risk assessment process.
2. An individual investigator or department may request an audit.
3. A project may be identified via an allegation of research misconduct or fraud or a suspected breach of regulations.
4. Projects may be selected at random. The Department of Health states that Trusts should be auditing a minimum of 10% of all research projects.
5. Projects may be randomly selected for audit by an external organisation.

Internal audits will be conducted by a sponsor's representative

14.2 Compliance

The CI will ensure that the trial is conducted in compliance with the principles of the Declaration of Helsinki (1996), and in accordance with all applicable regulatory requirements including but not limited to the Research Governance Framework, GCP, Trust and Research Office policies and procedures and any subsequent amendments.

14.3 Non-Compliance

Definition - A noted systematic lack of both the CI and the study staff adhering to the principles of the Declaration of Helsinki (1996), applicable regulatory requirements including but not limited to the Research Governance Framework, GCP, Trust and Research Office policies and procedures and any subsequent amendments, which leads to prolonged collection of deviations, breaches or suspected fraud.

These non-compliances may be captured from a variety of different sources including monitoring visits, CRFs, communications and updates. The sponsor will maintain a log of the non-compliances to ascertain if there are any trends developing or escalating. The sponsor will assess the non-compliances and action a timeframe in

which they need to be dealt with. Each action will be given a different timeframe dependent on the severity. If the actions are not dealt with accordingly, the sponsor will agree an appropriate action, including an on-site audit.

15. FINANCE AND FUNDING

The trial has been funded by the Barts Charity.

16. INDEMNITY

Queen Mary, University of London will act as a Sponsor, as defined by the Research Governance Framework for Health and Social Care (April 2005) for the project. The project will also be covered by the sponsor's insurance brokers on a "No Faults Compensation for Clinical Trials and/or Human Volunteer Studies". This policy will indemnify/cover the insured in respect of their legal liabilities arising out of the insured's activities.

17. DISSEMINATION OF RESEARCH FINDINGS

A meeting will be held after the end of the trial to allow discussion of the main results among the collaborators prior to publication. The success of the trial depends entirely on the wholehearted collaboration of a large number of doctors, nurses and community pharmacists. For this reason, chief credit for the main results will be given not to the committees or central organisers but to all those who have collaborated in the trial. A writing committee will be convened to produce publications on behalf of the CATPULT Steering Group, they will not be permitted to publish data obtained from participants in the CATAPULT trial that use study outcome measures without discussion with the Chief Investigator and/or the Trial Steering Committee. Only anonymized data will be used for dissemination of research findings.

18. REFERENCES

1. Dye, C., et al., *Consensus statement. Global burden of tuberculosis: estimated incidence, prevalence, and mortality by country. WHO Global Surveillance and Monitoring Project.* JAMA, 1999. **282**(7): p. 677-686.
2. **(ECDC, E.C.f.D.a.C.** TB situation in the EU/EEA 2012 [cited 2012 2012]; Available from: http://www.ecdc.europa.eu/en/healthtopics/tuberculosis/epidemiological_data/pages/tuberculosis_surveillance_europe.aspx.
3. Abubakar, I., et al., *Tuberculosis in the UK--time to regain control.* BMJ, 2011. **343**: p. d4281.
4. Stagg, H.R., et al., *Treatment of latent tuberculosis infection: a network meta-analysis.* Ann Intern Med, 2014. **161**(6): p. 419-28.
5. Fiske, C.T., et al., *Risk factors for treatment default in close contacts with latent tuberculous infection.* Int J Tuberc Lung Dis, 2014. **18**(4): p. 421-7.
6. Lein, A.D. and C.F. Von Reyn, *In vitro cellular and cytokine responses to mycobacterial antigens: application to diagnosis of tuberculosis infection and assessment of response to mycobacterial vaccines.* Am J Med Sci, 1997. **313**(6): p. 364-371.
7. Shieh, F.K., et al., *Predicting non-completion of treatment for latent tuberculous infection: a prospective survey.* Am J Respir Crit Care Med, 2006. **174**(6): p. 717-21.
8. Griffiths, C., et al., *Educational outreach to promote screening for tuberculosis in primary care: a cluster randomised controlled trial.* Lancet, 2007. **369**(9572): p. 1528-34.
9. Rennie, T.W., et al., *Patient choice promotes adherence in preventive treatment for latent tuberculosis.* Eur Respir J, 2007. **30**(4): p. 728-35.
10. Hirsch-Moverman, Y., et al., *Can a peer-based intervention impact adherence to the treatment of latent tuberculous infection?* Int J Tuberc Lung Dis, 2013. **17**(9): p. 1178-85.
11. Kominski, G.F., et al., *Costs and cost-effectiveness of adolescent compliance with treatment for latent tuberculosis infection: results from a randomized trial.* J Adolesc Health, 2007. **40**(1): p. 61-8.
12. Hirsch-Moverman, Y., et al., *Adherence to treatment for latent tuberculosis infection: systematic review of studies in the US and Canada.* Int J Tuberc Lung Dis, 2008. **12**(11): p. 1235-54.
13. Chang, A.H., A. Polesky, and G. Bhatia, *House calls by community health workers and public health nurses to improve adherence to isoniazid monotherapy for latent tuberculosis infection: a retrospective study.* BMC Public Health, 2013. **13**: p. 894.
14. Morano, J.P., et al., *Latent tuberculosis infection: screening and treatment in an urban setting.* J Community Health, 2013. **38**(5): p. 941-50.
15. Last, J.P. and J.M. Kozakiewicz, *Development of a pharmacist-managed latent tuberculosis clinic.* Am J Health Syst Pharm, 2009. **66**(17): p. 1522-3.
16. Taylor, K., *Asylum seekers, refugees, and the politics of access to health care: a UK perspective.* Br J Gen Pract, 2009. **59**(567): p. 765-72.
17. Sebastian, M.S. and G.H. Bothamley, *Tuberculosis preventive therapy: perspective from a multi-ethnic community.* Respir Med, 2000. **94**(7): p. 648-53.

18. Cheraghi-Sohi, S., et al., *What patients want from primary care consultations: a discrete choice experiment to identify patients' priorities.* Ann Fam Med, 2008. **6**(2): p. 107-15.
19. Jimenez-Fuentes, M.A., et al., *Rifampicin plus isoniazid for the prevention of tuberculosis in an immigrant population.* Int J Tuberc Lung Dis, 2013. **17**(3): p. 326-32.
20. Kunst, H. and K.S. Khan, *Age-related risk of hepatotoxicity in the treatment of latent tuberculosis infection: a systematic review.* Int J Tuberc Lung Dis, 2010. **14**(11): p. 1374-81.
21. Li, J., et al., *Adherence to treatment of latent tuberculosis infection in a clinical population in New York City.* Int J Infect Dis, 2010. **14**(4): p. e292-7.
22. Pareek, M., et al., *Screening of immigrants in the UK for imported latent tuberculosis: a multicentre cohort study and cost-effectiveness analysis.* Lancet Infect.Dis., 2011. **11**(6): p. 435-444.
23. Nicolau, I., et al., *Point-of-care urine tests for smoking status and isoniazid treatment monitoring in adult patients.* PLoS One, 2012. **7**(9): p. e45913.
24. Guerra, R.L., et al., *Point-of-care Arkansas method for measuring adherence to treatment with isoniazid.* Respir Med, 2010. **104**(5): p. 754-7.
25. van der Kop, M.L., et al., *The effect of weekly text-message communication on treatment completion among patients with latent tuberculosis infection: study protocol for a randomised controlled trial (WelTel LTBI).* BMJ Open, 2014. **4**(4): p. e004362.
26. Page, K.R., et al., *Improved adherence and less toxicity with rifampin vs isoniazid for treatment of latent tuberculosis: a retrospective study.* Arch Intern Med, 2006. **166**(17): p. 1863-70.
27. Leber, W et al., *Promotion of rapid testing for HIV in primary care (RHIVA2):a cluster-randomised controlled trial. The Lancet HIV*, 2015 **2**(6), pp.e229–e235

19. APPENDICIES

Appendix 1 – Information with regards to Safety Reporting in Non-CTIMP Research

	Who	When	How	To Whom
**SUSAR**	Chief Investigator	Report to the Sponsor, and QA manager within 24 hours<br>MREC within 15 days of learning of the event	SAE Report form for Non-CTIMPs, available from NRES website.	Sponsor and MREC
**Urgent Safety Measures**	Chief Investigator	Contact the Sponsor and MREC Immediately<br><br>Within 3 days	By phone<br><br>Substantial amendment form giving notice in writing setting out the reasons for the urgent safety measures and the plan for future action.	Main REC and Sponsor<br><br>Main REC with a copy also sent to the sponsor. The MREC will acknowledge this within 30 days of receipt.
**Progress Reports**	Chief Investigator	Annually (starting 12 months after the date of favourable opinion)	Annual Progress Report Form (non-CTIMPs) available from the NRES website	Main REC and Sponsor
**Declaration of the conclusion or early termination of the study**	Chief Investigator	Within 90 days (conclusion)<br><br>Within 15 days (early termination)<br><br>*The end of study should be defined in the protocol*	End of Study Declaration form available from the NRES website	Main REC with a copy to be sent to the sponsor
**Summary of final Report**	Chief Investigator	Within one year of conclusion of the Research	Where the study has met its objectives, the main findings and arrangements	Main REC with a copy to be sent to the sponsor

			for publication or dissemination including feedback to participants	

Appendix 2

Can latent tuberculosis infection in recent migrants be treated effectively and safely in primary care? A cluster randomised controlled trial

Short Title/Acronym : CATAPULT (Completion and Acceptability of Treatment Across Primary care and The commUnity for Latent Tuberculosis)

Latest protocol version: V7 01/05/2019
(though minor aspects of this plan reflect minor amendments to the protocol to be submitted shortly for approval)
ClinicalTrials.gov Identifier: NCT03069807

STATISTICAL ANALYSIS PLAN (SAP)

VERSION 1.0, 13TH AUGUST 2019

**Authorised by:**	**Signature**	**Date**
Dr Heinke Kunst, Chief Investigator<br>Email: h.kunst@qmul.ac.uk	Heinke Kunst	16/08/2019
Dr Andrew Copas, Trial Statistician<br>Email: a.copas@ucl.ac.uk	Andrew Copas	03/09/2019

Table of contents

1 ABBREVIATIONS 3

2 ABSTRACT – BACKGROUND AND DESIGN 4

3 OUTCOME MEASURES 6

3.1 Primary outcome 6
3.2 Secondary outcomes 6

4 DATA 7

4.1 CRF and variables 7
4.2 Data verification and lock 7
4.3 Data coding 8

5 SAMPLE SIZE ESTIMATION 9

6 ANALYSIS PRINCIPLES 9

6.1 Intention-to-treat (ITT) or per-protocol or other analysis population? 9
6.2 Significance level of tests 9
6.3 Baseline comparability 9
6.4 Adjustment for design factors 9
6.5 Losses to follow-up: handling missing data 10
6.6 Summarising models 10

7 ANALYSIS DETAILS 10

7.1 Recruitment and follow-up patterns 10
7.2 Baseline Characteristics 10
7.3 Intervention uptake 9
7.4 Analysis methods 10
7.4.1 Primary outcomes 10
7.4.2 Secondary outcomes 10
7.5 Sensitivity analyses 10
7.6 Subgroup analyses 10
7.7 Adjustment for baseline factors in analysis
7.8 Regression diagnostics 10
7.9 Multiple imputation by chained equations (MICE) 11

8 TABLES AND GRAPHS 11

8.1 Tables 12
8.2 Graphs 13

9 REFERENCES 13

Appendix: Calculation of primary outcome according to available data

1 ABBREVIATIONS

Acronyms	Meaning
CI	Confidence Interval
CRF	Clinical Report Form (questionnaire)
CXR	Chest X-ray
GP	General practitioner (primary care physician)
IGRA	Interferon gamma release assay
ITT	Intention-To-Treat
LFT	Liver function test
LTBI	Latent tuberculosis infection
MAR	Missing At Random
MI	Multiple Imputation
MITT	Modified Intention-to-Treat
MICE	Multiple Imputation by Chained Equations
OR	Odds Ratio
SAP	Statistical Analysis Plan
TB	Tuberculosis
TMF	Trial Master File

2 ABSTRACT – BACKGROUND AND DESIGN

Objectives: Primary objective

- To determine whether an innovative programme of treating latent tuberculosis infection (LTBI) in primary care increases LTBI treatment completion (defined as taking at least 90% of antibiotic dosages based on pill count having started) compared with current practice (treatment in secondary care).

Secondary objectives

- To assess the proportion of patients completing less than 80%, 80-89.9% and 90% or more of antibiotic dosages based on pill count.
- To assess the proportion of patients who are adherent to treatment based on the MARS5 tool, prescription collection and point-of-care urine testing for metabolites of Isoniazid.
- To describe the proportion of individuals in the two treatment arms who accept LTBI treatment.
- To assess the incidence of adverse effects of treatment for LTBI including drug induced liver injury (DILI) in both arms.
- To assess rates of active tuberculosis (TB) cases in primary and secondary care during and after the study period measured as case notifications to Public Health England (PHE) using the Enhanced TB surveillance (ETS) database.
- To assess patient satisfaction of treatment received in primary care compared with secondary care.
- To evaluate cost effectiveness of LTBI treatment in primary care compared with treatment in secondary care.

Population studied:

Inclusion criteria:

Practice level: GP surgeries within Newham

Individual level: Patients with LTBI aged 16-35 and who have entered the UK less than 5 years ago from a country with a TB incidence of greater than 150/100,000 or sub-Saharan Africa. In this trial LTBI is defined as a positive IGRA test without any symptoms or physical signs of active TB and no evidence of active TB on Chest X-ray.

Exclusion criteria:

Individual level: Patients who do not meet the criteria for the standard Newham primary care treatment protocol will be excluded which include:

1) Pregnant or breastfeeding women.

2) Patients requiring medications that cannot be safely taken with Rifinah

3) HIV infection.

4) Individuals with known liver disease, or abnormal liver function tests (LFTs) as defined in the Newham liver function protocol.

5) Diagnosis of cirrhosis (jaundice, hematemesis, ascites or previous episodes of liver encephalopathy).

6) Chronic or active hepatitis B or hepatitis C virus infection.

7) Previous treatment for TB or LTBI.

8) Individuals who are unable to consent or who would usually be offered LTBI treatment under DOT because of their mental or social disabilities or those with drug or alcohol abuse.

9) Evidence of active TB (based on history, examination, blood tests and/or Chest X-ray finding).

Trial design, intervention and control conditions:

This is a cluster randomised, parallel group, superiority trial.

The trial intervention is the setting of LTBI Treatment: either in the community, managed by the GP and community pharmacist or in secondary care, managed by the TB doctor and nurse. All eligible patients (those aged 16-35 and who have entered the UK less than 5 years ago from a country with a TB incidence of greater than 150/100,000, or sub-Saharan Africa) are offered screening for LTBI in the form of an IGRA and other blood tests when they register to join a GP practice as per routine care within Newham. All patients eligible for screening will be given a Patient Information Sheet explaining the study. Patients with a positive IGRA not meeting any of the exclusion criteria will be managed in different ways between intervention and control arms.

In the intervention (primary care) arm patients are invited to see the GP and at this review, the GP will take a brief history and perform a physical examination, to exclude a diagnosis of active TB. The patient will also be asked to complete a questionnaire about their understanding of LTBI. If there is no evidence of active TB, the GP will explain to the patient the diagnosis of LTBI, and offer the patient treatment pending a Chest X-ray (CXR). The patient will then attend local radiology services for the CXR, and the GP will be sent the result. If the CXR shows no signs of active TB, the patient is eligible for the study and to start treatment. The GP will generate an electronic prescription that can be collected from community pharmacies participating in the trial. The patient will be asked to attend a listed pharmacy to start treatment, and this will also be the source of further medication, testing of LFT and data collection.

In the control (secondary care) arm, patients will be informed that they are being referred to secondary care (TB Clinic) for review. At this review, the TB doctor will take a brief history, perform a physical examination, and organise a CXR to exclude a diagnosis of active TB. If there is no evidence of active TB, the TB doctor will explain the diagnosis of LTBI, and offer the patient treatment. The patient will also be asked to complete a brief questionnaire about their knowledge of LTBI. The patient will be given a 1-month supply of medication. The TB nurse and the clinic is the source of further medication, testing of LFT and data collection.

In both study arms further supply of medication is dependent on satisfactory LFT results and the absence of adverse events as recorded at monthly visits for further medication supply. At each monthly visit data on adherence is collected. There are three monthly follow-up visits reflecting that the intended full treatment duration is 84 days. At the 2-month follow-up visit a patient satisfaction questionnaire is administered.

Sample size:

At least 20 practices required and a number of individuals dependent on the number of practices – see section 5.1 for further information.

Randomisation:

This was conducted in phases according to when GP practices gave consent and also as further practices were recruited in response to slower than expected recruitment of individuals within practices. In the first phase 20 practices were stratified by the cross-classification of two binary factors based on practice size and number of IGRA positives identified previously. In three further phases of randomisation practices 21-22, 23-29 and 30-34 were randomised. Of these the randomisation of practices 23-29 was stratified by the number of IGRA positives and in the other phases randomisation was unrestricted. For the third phase as 7 practices were randomised the allocation was 3 to one study arm 4 to the other (determined at random). In the final phase the allocation was 2 to one study arm and 3 to the other, allocating 3 to the arm that was allocated only 3 in the third phase, to ensure an overall 1:1 allocation of 15 practices to each arm. At all phases and within all strata as applicable randomisation was through random permutation using Stata software. Randomisation was conducted by the trial statistician.

Blinding:

Data collection and analysis will be unblinded due to the nature of the intervention, cluster level randomisation and different data collection systems for the two study arms.

3 OUTCOME MEASURES

The measures and details of how collected where not obvious are as follows:

3.1 Primary Outcome

Completion of at least 90% of prescribed therapy as assessed by pill count (taking more than 76 out of 84 dosages) having accepted treatment. Pill counts will be recorded monthly, where a patient fails to bring their medication information will be obtained by patient report. Treatment completion will be assessed at the end of treatment by the community pharmacist in primary care and the TB nurse in secondary care. To avoid bias, an independent blinded researcher will verify treatment completion where there is uncertainty. For clarity, patients who stop treatment even following guidance after an adverse event are considered to have not completed treatment. Patients who are not known to have stopped treatment but do not attend (or complete by phone) the final follow-up visit will be considered to have missing data for the primary outcome.

See Appendix for details of how the primary outcome is derived.

3.2 Secondary Outcomes

- Treatment completion rate (defined as for primary outcome) reported as <80, 80-90, 90+% of antibiotic dosages (considered an ordinal outcome – treatment not completed, partially completed, completed)

- Treatment adherence as assessed using the five-point adherence questionnaire (MARS5 tool), which is asked at each follow-up visit. This will be summarised as an ordinal outcome: treatment stopped (at least one prescription missed), poor adherence at 2 or 3 visits, poor adherence at one visit, good adherence at all visits (or all visits where questionnaire completed). The definition of poor adherence will be data driven – we anticipate a bimodal distribution of MARS5 scores – but decided before analysing by study arm. In the event however of a highly skewed distribution where almost all (>95% participant visits) completing the tool have a score >90% of the maximum, indicating 'good' adherence, then a score of less than <90% of maximum will be treated as 'poor adherence' for the purpose of descriptive reporting by trial arm but there will be no statistical testing for this outcome.
- Treatment adherence as assessed by urine testing for metabolites of Isoniazid, which is typically done two or three times during follow-up. This will be summarised as an ordinal outcome: treatment stopped (at least one prescription missed), two or more negative tests (no metabolites detected), one negative test, all tests positive.
- Treatment acceptance defined as proportion initiating treatment and attending TB clinics or community pharmacies (as per study arm) on at least one occasion. This is defined as a proportion from two different 'denominators' separately. Firstly, from all recruited individuals with LTBI aged 16-35 and who have entered the UK within the last 5 years from a country with a TB incidence of greater than 150/100,000 or sub-Saharan Africa that are eligible for treatment. Secondly, at a practice level, from amongst all IGRA positive individuals aged 16-35 and who have entered the UK less than 5 years ago from a country with a TB incidence of greater than 150/100,000 or sub-Saharan Africa whilst the practice is recruiting to CATAPULT. The comparison between arms under the second denominator is informal and will not involve any statistical testing.
- Adverse events on treatment, including DILI, leading to discontinuation of treatment or hospitalisation (note the safety of treatment is assessed every month by the pharmacist in primary care and by the TB nurse in secondary care).
- Active TB occurring within 2 years after enrolment. This will be performed through matching the study population with the national Enhanced TB Surveillance System, where information on all reported TB cases nationally are recorded, and follow-up for each individual is censored at two years from enrolment or at the date of data extraction whichever is sooner. Subject to timely data permissions and extraction this outcome will be reported in the main trials results paper.
- Assessment of patient satisfaction using a single summary response item in a standardised treatment satisfaction questionnaire after two months of treatment.
- Evaluation of cost-effectiveness of treatment in primary care compared to secondary care (not considered further in this plan)

4 DATA

4.1 CRF and variables

Full details of data collection and timing are described in the trial protocol. Basic data on age, country of birth, socioeconomic status, substance misuse and pre-existing medical conditions (such as diabetes) will be recorded in all patients electronically in primary care using EMIS. Information on adherence and adverse effects will be obtained every month and recorded electronically by Community Pharmacists and in TB Clinic. See below for summary of main data collection tools and timing.

	Day 0	Day 15	Day 30	Day 60	Day 90
Questionnaire (Understanding and knowledge of LTBI)	X				
Blood tests for LFTs		X			
Mars- 5 tool questionnaire / pill count			X	X	X
Questionnaire (adherence/ adverse effects)			X	X	X
Urine test (Colour and INH metabolites)			X	X	X
Patient satisfaction questionnaire				X	

4.2 Anonymisation, database verification and lock

Before data are sent to the study statistician a unique numerical study identifier will be created for each trial participant and serve to enable the different study datasets to be linked. A dataset of study identifiers and corresponding NHS numbers will be retained within the trial clinical team to facilitate the checking of data, but NHS number and all other potentially identifying information will not be shared with the trial statistician under any circumstances.

At the end of the study a copy of each dataset will be passed to the trial statistician for checking. Basic data checks will be performed by the trial statistician including checks of range, consistency and missing data.

Any problems with trial data will be queried with the Trial Manager or Data Manager as appropriate. If possible, data queries will be resolved; although it is accepted that due to administrative reasons and data availability a small number of problems will continue to exist. These will be minimised.

The datasets will then be locked and the locked versions supplied to the statistician for final analysis.

4.3 Data coding

Details of the variables, including variable coding lists will be provided from all the databases to support the analysis.

5 SAMPLE SIZE ESTIMATION

From published data and our retrospective review, 70% of patients currently complete LTBI treatment. We expect treatment completion in primary care to improve by 15% compared with secondary care (from 70% to 85%). To detect this difference with 80% power, and 5% significance level, an individually randomised trial would require 268 participants. We planned to conduct our trial in a minimum of 20 GP practices (10 intervention and 10 control), noting that as the number of GP practices increases, the required total sample size of individual participants falls. With 20 practices, we would have needed to adjust for the effect of clustering by increasing the sample size to 780 participants (or 39 patients per practice) assuming an ICC of 0.05 (Griffiths Lancet 2007). To allow for loss to follow up and treatment non-acceptance we would have inflated the required sample size by 30% to 1014 participants (51 per GP practice). The final number of practices randomised was 34, though as 14 were randomized after the initial phase we expect variability in cluster size reflected by a coefficient of variation of 0.5. Under the same assumptions as before the number of individuals required providing the primary outcome is 442 (221 per arm), inflated due to loss to follow up and treatment non-acceptance to 576 (17 per practice)

The trial is not powered to detect differences between the study arms for any of the other secondary outcomes.

6 ANALYSIS PRINCIPLES

6.1 Intention-to-treat (ITT) or per-protocol or other analysis population?

All analyses will be conducted on an intention-to-treat basis. We will include all eligible participants regardless of how well the GP practice followed the study protocol or how well the participant complied with their treatment plan. We will exclude any patients however who have withdrawn from the trial and specified they do not wish their data to be analysed.

Some outcomes are only applicable for certain subgroups. The primary outcome is defined only for patients who accept treatment. Patient satisfaction is only defined for patients who are still under care at 2 months, when the satisfaction questionnaire is administered. Treatment acceptance is defined as a proportion from two different 'denominators' separately. Firstly the outcome is defined from amongst all individuals with LTBI who are recruited to the trial and are known eligible for treatment. This outcome is then also defined (at a practice level) from amongst all IGRA positive individuals whilst the practice is recruiting to CATAPULT. See section 3.2 for more detail.

6.2 Significance level of tests

All confidence intervals will be 95% and two-sided. Statistical tests will use a two-sided *p* value of 0.05.

6.3 Baseline comparability

Baseline characteristics will be summarised by randomised group.

6.4 Adjustment for design factors

We will adjust the analyses for practice size and number of previous IGRA positives prior to recruitment to trial, our stratification factors.

6.5 Losses to follow-up: handling missing data

There may be missing data for the primary outcome arising for patients who attend follow-up visits after one and two months, and collect prescriptions, but miss the final visit. For such individuals we will impute the outcome (see 7.9 for details) as our primary approach, and will also as a 'sensitivity analysis' consider all such individuals to have failed to complete treatment.

6.6 Summarising models

Wherever possible, analysis of outcomes will involve a parametric model. Treatment effect estimates will be presented as regression coefficients and 95% confidence intervals. Binary logistic regression will be used for binary outcomes, ordinal logistic regression for ordinal outcomes such as satisfaction with care, or adherence measured by Mars-5 or urine tests. Adjusted effect measures are considered the primary effect measures, though unadjusted are also reported for completeness. All regression models fitted will include a random effect for GP practice.

7 ANALYSIS DETAILS

7.1 Recruitment and follow-up patterns

The number of patients who were lost to follow-up (withdrawn by patient choice) will be reported by randomisation group and presented in the CONSORT diagram. The number of patients recruited will be presented by clinic, graphically.

7.2 Baseline Characteristics

Baseline characteristics will be reported for each of the two randomisation arms, including knowledge of TB. Summary measures for the baseline characteristics of each arm will be presented as mean and standard deviation for continuous (approximately) normally distributed variables, medians and interquartile ranges for non-normally distributed variables, and frequencies and percentages for categorical variables.

7.3 Intervention uptake

Since the primary and secondary outcomes include measures of 'intervention uptake' no further outcomes are required to describe this aspect of the trial.

7.4 Analysis Methods

7.4.1 Primary outcomes

The primary outcome will be analysed using a logistic regression model. Results will be reported as odds ratios (OR) for the intervention compared to control with their corresponding 95% confidence intervals (CI) and two-sided p-values. The ICC for the primary outcome will be reported.

7.4.2 Secondary outcomes

As stated earlier, the trial has not been powered to detect differences between the randomised arms for any of the secondary endpoints. As for the primary analysis, secondary analyses will be based on comparison of two randomisation arms.

7.5 Sensitivity analyses

A sensitivity analysis will be conducted in which those with missing data in the primary outcome will be assumed to have failed to complete treatment.

7.6 Subgroup and stratified analyses

The analysis of TB incidence comparing intervention and control groups will be stratified by those who did or did not accept treatment, and if accepted by whether treatment was completed. None other subgroup analyses are planned.

7.7 Adjustment for baseline factors in analysis

Besides adjusting for the stratification factors (practice size and number of IGRA positives prior to recruitment) we shall also adjust for other baseline factors. These factors are participants' age, sex, country of birth, years in the UK, TB risk factors, and TB knowledge (subject to satisfactory data completeness). In the event of collinearity or small 'cell size' for any of these factors then we shall consider combining categories to improve stability. Any decisions to combine categories will be made before beginning the analysis of the intervention effect on the primary outcome. Adjustment will be made for the same factors for all outcomes. The adjusted effect measures are considered the primary effect measures for all outcomes.

7.8 Regression diagnostics

For outcomes where we use ordinal logistic regression the distribution of each outcome will be informally compared between arms to assess whether the assumption of proportional odds is qualitatively appropriate. If the assumption is not appropriate we shall consider conducting two separate logistic regressions at different thresholds of the ordinal measure to represent the effect of the intervention.

7.9 Multiple imputation by chained equations (MICE)

We plan to impute the primary outcome for individuals where this is missing. The function "mi" impute will be used in Stata for the MICE method. Imputation will be conducted separately by study arm, and the imputation model will be fitted only to those who attended the preceding visit and collected that prescription. A logistic regression model will be used to impute the binary outcome directly (not the underlying continuous measure of completion) based on completion related measures and adverse effects, specifically (i) the count of remaining pills (observed or self-reported if pills not brought to visit) at two months and (ii) the count of remaining pills at one month, and (iii) reporting of moderate or severe adverse events in any of the defined classes that are asked at follow-up visits at one or two months. In the event that the imputation model does not converge (in either study arm) due to small cell size we will attempt to fit a reduced model including as many of the three predictive factors above as possible prioritising them in the order listed.

8 TABLES AND GRAPHS:

8.1 Tables

Table 1: Baseline characteristics of the participants

Variable / Groups		Intervention<br>N=<br>%	Control<br>N=<br>%
**Socio-demographic factors**			
Age	Median [IQR], years		
Sex			
Country of birth			
Years in the UK			

Table 2: Effect of intervention on outcome measures

Outcome measures	Intervention % (N)	Control % (N)	OR (CI)	Adjusted OR (CI)
**Primary outcome**				
Etc.				

8.2 Graphs

Graph 1: CONSORT diagram including follow up rates at 1, 2 and 3 months by arm
Graph 2: Plot of primary outcome by GP clinic, grouped by arm, to demonstrate variability between clinics
Graph 2: Bar chart of satisfaction measures and Mars-5 score by intervention arm

9 REFERENCES

Appendix: Calculation of primary outcome according to available data

The primary outcome is defined giving emphasis to prescription collection and pill count data but using self-reported adherence where pill count is unavailable. Pill count data applies to all time up till that visit - we assume patients do not throw away pills so pill count gives a cumulative summary to that point. Also, the dates of follow-up visits are set so that the individual typically attends around the time at which the next treatment pack should be completed. Consequently, the outcome can be derived using only data from the final visit. This gives rise to the following hierarchical approach to deriving the outcome:

1. If a patient stops treatment by patient choice or due to adverse event, failing to attend from one or two months onwards, or to collect the required prescriptions, then their adherence is poor (<80%)
2. If a patient brings pills to final visit and visit is “on time or late” relative to date when treatment pack would be completed, then then adherence is (number of doses taken) / (total number of doses prescribed: 84)
3. If a patient brings pills to final visit and visit is “early” then adherence is (number of doses taken / (number of doses intended to have been taken by that day as determined at previous visit)
4. If a patient does not bring pills to final visit and has not stopped treatment then adherence is self-reported and the direct question is “how many did you miss over the last month”, with response options 0, 1-3, 4-5, 6-8, 9 or more. This is treated as a pill count and the approach taken at step 2 or 3 above is performed according to whether the visit is early, on time or late. Note that it is not possible to calculate an exact percentage completion since the missed doses are reported in categories. Those reporting 0, 1-3, 4-5, or 6-8 missed doses will be considered >90% complete unless the final visit is very early. Those reporting 9 or more missed doses will be considered 80-90% complete.
5. If a patient does not attend their final visit but has not stopped treatment (has collected final prescription) then they are missing for the primary outcome.
