# Supplementary material for: Treatment of latent tuberculosis infection in migrants in primary care versus secondary care
Source: Eur Respir J. 2024 Nov 7;64(5):2301733. doi: 10.1183/13993003.01733-2023 (PMC11540984; doi:10.1183/13993003.01733-2023)
Supplement: Supplementary file 2 [file ERJ-01733-2023.Shareable.pdf]

SHAREABLE PDF

EUROPEAN RESPIRATORY JOURNAL
ORIGINAL RESEARCH ARTICLE
M. BURMAN ET AL.

Treatment of latent tuberculosis infection in migrants in primary care *versus* secondary care

Matthew Burman, Dominik Zenner, Andrew J. Copas, Lara Goscé, Hassan Haghparast-Bidgoli, Peter J. White, Vicky Hickson, Opal Greyson, Duncan Trathen, Richard Ashcroft, Adrian R. Martineau, Ibrahim Abubakar, Christopher J. Griffiths and Heinke Kunst

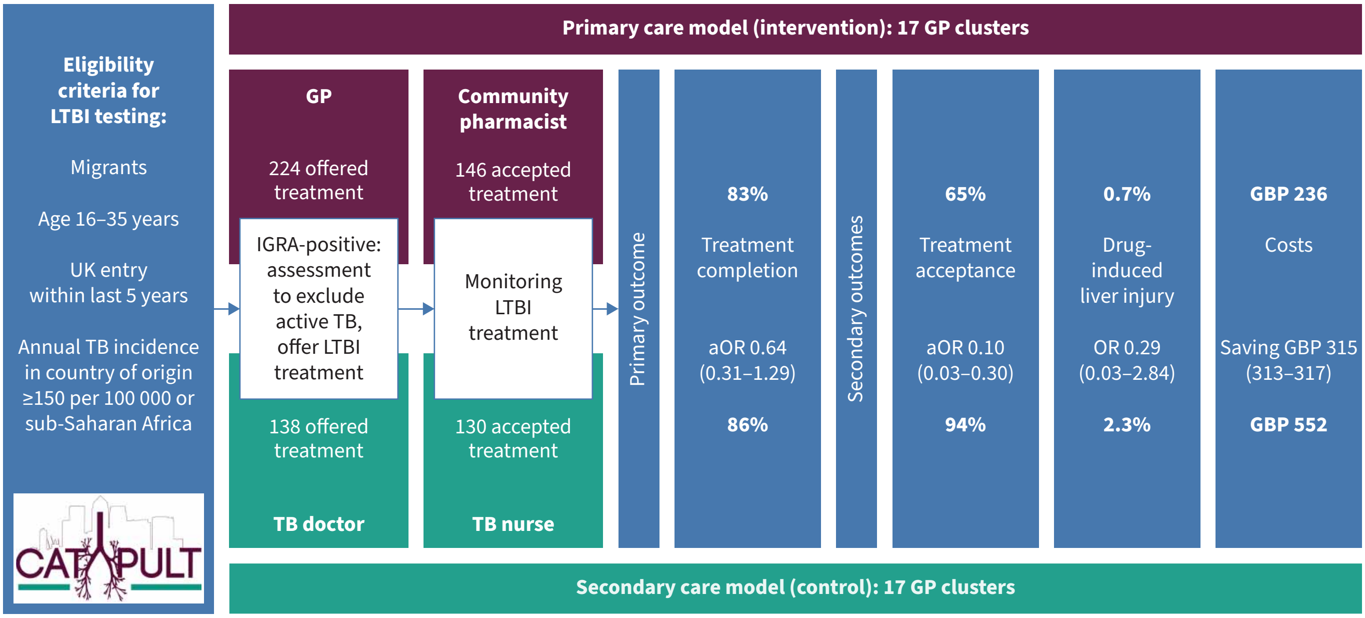

GRAPHICAL ABSTRACT CATAPuLT (Completion and Acceptability of Treatment Across Primary care and the commUnity for Latent Tuberculosis) trial showing the eligibility criteria, trial design and results. 95% confidence intervals are indicated in brackets. LTBI: latent tuberculosis infection; TB: tuberculosis; GP: general practitioner; IGRA: interferon-γ release assay.

EUROPEAN RESPIRATORY JOURNAL
ORIGINAL RESEARCH ARTICLE
M. BURMAN ET AL.

SHAREABLE PDF

Treatment of latent tuberculosis infection in migrants in primary care *versus* secondary care

Matthew Burman[1,2], Dominik Zenner[1], Andrew J. Copas[3], Lara Goscé[3], Hassan Haghparast-Bidgoli[3], Peter J. White[4,5], Vicky Hickson[1], Opal Greyson[1], Duncan Trathen[6], Richard Ashcroft[7], Adrian R. Martineau[1], Ibrahim Abubakar[3], Christopher J. Griffiths[1] and Heinke Kunst[8,9]

[1]Wolfson Institute of Population Health, Barts and The London School of Medicine and Dentistry, Queen Mary University of London, London, UK. [2]Homerton Healthcare NHS Foundation Trust, London, UK. [3]Institute for Global Health, University College London, London, UK. [4]MRC Centre for Global Infectious Disease Analysis and NIHR Health Protection Research Unit in Modelling and Health Economics, Department of Infectious Disease Epidemiology, Imperial College London, London, UK. [5]Modelling and Economics Unit, UK Health Security Agency, London, UK. [6]Newham Clinical Commissioning Group, London, UK. [7]City Law School, City, University of London, London, UK. [8]Blizard Institute, Barts and The London School of Medicine and Dentistry, Queen Mary University of London, London, UK. [9]Barts Health NHS Trust, London, UK.

Corresponding author: Heinke Kunst (h.kunst@qmul.ac.uk)

Check for updates

Shareable abstract (@ERSpublications)
This cluster-randomised control trial found that the treatment of latent tuberculosis infection in recent arrivals to the UK can be safely and effectively managed within primary care when compared to specialist secondary care services at lower cost. https://bit.ly/4cmKzKm

Cite this article as: Burman M, Zenner D, Copas AJ, *et al*. Treatment of latent tuberculosis infection in migrants in primary care *versus* secondary care. *Eur Respir J* 2024; 64: 2301733 [DOI: 10.1183/13993003.01733-2023].

This extracted version can be shared freely online.

Copyright ©The authors 2024.

This version is distributed under the terms of the Creative Commons Attribution Licence 4.0.

This article has an editorial commentary: https://doi.org/10.1183/13993003.01569-2024

Received: 9 Oct 2023
Accepted: 31 July 2024

Abstract

Background Control of latent tuberculosis infection (LTBI) is a priority in the World Health Organization strategy to eliminate TB. Many high-income, low TB incidence countries have prioritised LTBI screening and treatment in recent migrants. We tested whether a novel model of care, based entirely within primary care, was effective and safe compared to secondary care.

Methods This was a pragmatic cluster-randomised, parallel group, superiority trial (ClinicalTrials.gov: NCT03069807) conducted in 34 general practices in London, UK, comparing LTBI treatment in recent migrants in primary care to secondary care. The primary outcome was treatment completion, defined as taking ⩾90% of antibiotic doses. Secondary outcomes included treatment acceptance, adherence, adverse effects, patient satisfaction, TB incidence and a cost-effectiveness analysis. Analyses were performed on an intention-to-treat basis.

Results Between September 2016 and May 2019, 362 recent migrants with LTBI were offered treatment and 276 accepted. Treatment completion was similar in primary and secondary care (82.6% *versus* 86.0%; adjusted OR (aOR) 0.64, 95% CI 0.31–1.29). There was no difference in drug-induced liver injury between primary and secondary care (0.7% *versus* 2.3%; aOR 0.29, 95% CI 0.03–2.84). Treatment acceptance was lower in primary care (65.2% (146/224) *versus* 94.2% (130/138); aOR 0.10, 95% CI 0.03–0.30). The estimated cost per patient completing treatment was lower in primary care, with an incremental saving of GBP 315.27 (95% CI 313.47–317.07).

Conclusions The treatment of LTBI in recent migrants within primary care does not result in higher rates of treatment completion but is safe and costs less when compared to secondary care.
